# Effects of *Saccharomyces cerevisiae* Postbiotic on Growth Performance, Selected Immune and Antioxidant Gene Expression, and Resistance to *Fusarium oxysporum* Infection in Nile Tilapia (*Oreochromis niloticus*)

**DOI:** 10.3390/biology15171445

**Published:** 2026-08-22

**Authors:** Nagwa Mohamed Abdou Romeih, Mahmoud Mostafa Mahmoud, Yosra M. I. El Sherry, Amna Mohamed Eltayeb, Amr Abdallah, Abeer M. Mahmoud, Marco Albano, Alaa G. M. Osman, Ebtsam Sayed Hassan Abdallah

**Affiliations:** 1 Department of Aquatic Animal Medicine and Management, Faculty of Veterinary Medicine, Assiut University, Assiut 71529, Egypt; nagwaromeih@aun.edu.eg (N.M.A.R.); ebtsamsayed@aun.edu.eg (E.S.H.A.); 2 Department of Aquatic Animal Medicine and Management, School of Veterinary Medicine, Badr University in Assiut, Assiut 71511, Egypt; 3 Department of Fish Diseases, Faculty of Veterinary Medicine, Aswan University, Sahari, Airport Way, Aswan 81528, Egypt; yosramohamed@vet.aswu.edu.eg; 4 Department of Aquatic Animal Medicine, Faculty of Veterinary Medicine, New Valley University, Kharga 72511, Egypt; amnaeltayeb@vet.nvu.edu.eg; 5 Department of Architectural Engineering, Imam Mohammad Ibn Saud Islamic University (IMSIU), Riyadh 11432, Saudi Arabia; asabdullah@imamu.edu.sa; 6 Division of Endocrinology, Department of Medicine, University of Illinois Chicago, Chicago, IL 60612, USA; 7 Department of Kinesiology and Nutrition, College of Applied Health Sciences, University of Illinois Chicago, Chicago, IL 60612, USA; 8 Department of Veterinary Sciences, University of Messina, Polo Universitario Dell’Annunziata, 98168 Messina, Italy; malbano@unime.it; 9 Department of Environmental and Fish Biology, Faculty of Science, Al-Azhar University, Assiut 71524, Egypt; agosman@azhar.edu.eg

**Keywords:** fermented product, growth performance, serum biochemical parameters, hepatic, splenic and intestinal gene expression, *Fusarium oxysporum* resistance, teleost

## Abstract

This study evaluated graded dietary doses of *Saccharomyces cerevisiae* postbiotic (0, 1.25, 2.5 and 5 g kg^−1^) on growth, feed utilization, serum biochemistry, expression of *IGF-1*, *SOD2*, *IL-1β*, *IL-10* and *IgM*, and resistance to *Fusarium oxysporum* in Nile tilapia (*Oreochromis niloticus*; 35.1 ± 0.9 g) after 30 and 60 days of feeding. Although the 5 g Kg^−1^ inclusion significantly increased WG (59.4 ± 0.9) and SGR (1.5 ± 0.0) and decreased FCR (0.8 ± 0.0), significant positive effects on multiple endpoints were also recorded at 1.25 and 2.5 g/kg. Serum total protein and globulin rose with increasing dose and feeding duration, while albumin showed limited change, lowering the A/G ratio. AST remained unchanged. Creatinine and glucose declined with postbiotic supplementation; the glucose reduction effect was dose-dependent, whereas creatinine was unaffected by feeding duration. The highest gene expression of hepatic *IGF-1*, *SOD2*, *IL-1β*, and *IL-10* and splenic *IL-1β*, *IL-10*, and *IgM* was in T3 at both checkpoints; however, the highest gene expression of intestinal *IL-1β*, *IL-10*, and *IgM* expression was in T1 at both time points. The pathogenicity experiment showed a significant survival against *F. oxysporum* (*p* < 0.0001) compared to controls. The results suggested that dietary *S. cerevisiae* postbiotic improved growth, biochemical and molecular responses, and survival of Nile tilapia against *F. oxysporum*.

## Simple Summary

Non-living microbial products called postbiotics have the potential to improve health without raising the same safety issues as live probiotics. The effects of varying *Saccharomyces cerevisiae* postbiotic levels in the diet of Nile tilapia over 60 days were evaluated for growth, feed utilization, gene expression, and disease resistance in this study after being fed 0, 1.25, 2.5, or 5 g/kg. It was found that dietary supplementation with 5 g/kg of *S. cerevisiae* postbiotic for a 60-day feeding period significantly improved Nile tilapia weight gain, specific growth, and feed conversion ratio. It improved serum biochemical parameters and modulated the expression levels of the liver, spleen, and intestine with a significant decrease in the susceptibility of Nile tilapia to *Fusarium oxysporum* infection. Overall, the dietary *S. cerevisiae* postbiotic enhanced growth and disease resistance in Nile tilapia.

## 1. Introduction

Aquaculture is an important resource for providing humans with a sufficient quantity of animal protein, since production from catch fisheries has been stagnant for the past few decades [1]. The freshwater fish Nile tilapia (*Oreochromis niloticus*), native to Africa, is currently farmed in numerous nations all over the world. Freshwater Nile tilapia farming in Egypt yielded 980,004 tons in 2024, with a corresponding value of approximately 1,168,235.77 (USD 1000) [2]. Egypt is currently the top tilapia producer in Africa, accounting for over 71% of the continent’s total production, and the third producer globally, behind China and Indonesia [3]. However, the rapid expansion and intensification of tilapia farming have resulted in environmental stressors and disease outbreaks [4], highlighting the need for sustainable strategies that improve fish health and immunity [4,5].

Within this framework, functional feed additives were developed. Feed additives contain immunomodulatory ingredients that regulate and balance immune function. They can both stimulate protective responses when needed and dampen excessive inflammation to maintain immune homeostasis, often by modulating the expression of both pro- and anti-inflammatory cytokines [6] such as those produced by postbiotics.

According to the International Scientific Association for Probiotics and Prebiotics (ISAPP) in 2021, a postbiotic was defined as “a preparation of inanimate microorganisms and/or their components that confers a health benefit on the host” [7]. It has many advantages, such as its long shelf life, safe dose limits, chemical structure, and end products of signaling molecules that exhibit various activities. They contain immunomodulatory bioactive metabolites such as short-chain fatty acids, bacteriocins, and organic acids, which are responsible for their antimicrobial, antioxidant, immunomodulatory, and anti-inflammatory activities [8]. They also provide positive physiological benefits, possibly tied to mucosal immunity, and the improvement of intestinal barrier function [9]. They target the host–microbe–pathogen interface, avoiding biotic and immune imbalances as well as inflammation [10]. This premise gives them potential applications as food additives and dietary supplements for aquatic and other organisms [11].

The majority of in vitro and in vivo research focuses on postbiotics derived from various *Lactobacillus* and *Bifidobacterium* strains, as well as *Saccharomyces species* [12]. *Saccharomyces cerevisiae* and its by-products promote growth, weight gain, and feed conversion ratio; increase lysozyme activity and serum total immunoglobulins; elevate innate immunological defenses; activate the complement system alternative and classical pathways, and improve overall health. These effects may be attributed to its immunostimulatory and bioactive properties [13]. *S. cerevisiae* contains bioactive β-glucans, mannan-oligosaccharides, and nucleotides. In addition, it is relatively high in protein, energy, and micronutrient contents, such as vitamins and trace elements [13,14].

Numerous investigations have confirmed that *S. cerevisiae* immune-saccharides improve the health of aquatic animals [15,16]. It was studied in several farmed freshwater fish species, including Nile tilapia, channel catfish (*Ictalurus punctatus*), rohu (*Labeo rohita*), jian carp (*Cyprinus carpio* var. Jian), Gibel carp (*Carassius gibelio*), common carp (*C. carpio*), and pacu (*Piaractus mesopotamicus*) [12,17,18,19,20,21], as well as marine fish such as rainbow trout (*Oncorhynchus mykiss*), turbot (*Scophthalmus maximus*), gilthead sea bream (*Sparus aurata*), largemouth bass (*Micropterus salmoides*), hybrid striped bass (*Morone chrysops* × *M. saxatilis*), Thai panga (*Pangasianodon hypopalmus* × *Pangasius bocourti*), and European seabass (*Dicentrarchus labrax*) [14,22,23,24,25,26,27,28].

*Saccharomyces cerevisiae* postbiotic enhanced disease resistance and survival rates against *Aeromonas hydrophila* in juvenile common carp and crucian carp (*C. auratus*), *A. hydrophila* NJ-1 and *A. veronii* HM091 in channel catfish [12], Columnaris disease in catfish [29], and *A. veronii biovar sobria* in Nile tilapia [30]. This could be explained by the fact that β-glucan (BG) constitutes approximately 50–60% of the polysaccharides present in the yeast cell wall. BG can bind to specific pathogen-associated molecular pattern receptors to induce an innate immune response [31]. β-Glucan immune stimulation modulates acute-phase response-related gene expression in the head kidney and liver of fish, whereby the liver responds to pathogens by producing additional acute-phase proteins [32]. The synthesis, secretion, and metabolism of xenobiotics are just a few of the physicochemical processes that the liver can regulate [33]. This has shown encouraging immunological activity, including phagocytosis, superoxide anion production, and lysozyme activity [34], thereby boosting fish growth and immune responses [35,36]. However, these benefits differ depending on the fish species, age, and the solubility and chemical composition of the yeast products [37]. The product used in this study is a fermentation product derived from *S. cerevisiae* culture (DVAQUA^®^, Diamond V Mills Inc., Cedar Rapids, IA, USA). It consists of inactivated yeast cells, cell wall components (β-glucans and mannan oligosaccharides), and a complex of bioactive metabolites generated during the fermentation process, including short-chain fatty acids, organic acids, peptides, nucleotides, vitamins, and antioxidants. These non-viable components are responsible for the immunomodulatory and health-promoting effects of the postbiotic [38].

Fungal infections in aquaculture represent a significantly underexplored but economically critical problem. Unlike bacterial pathogens, such as *Aeromonas* sp. and *Streptococcus* sp., fungal infections have received minimal research attention despite their increasing prevalence and severity in aquaculture systems globally. *Fusarium oxysporum* is a versatile filamentous fungus that has emerged as an important pathogen in aquaculture environments. *Fusarium* species have been documented in several aquatic animals and amphibians [39,40], including Nile tilapia [41]. *Fusarium oxysporum* species complex (FOSC) infections have been documented in zebrafish (*Danio rerio*) farming facilities, where the fungus forms white mycelial growth on tank surfaces and infiltrates fish muscle tissue [42]. 

Although *F. oxysporum* infections in fish have been reported and well documented, studies testing the efficacy of *S. cerevisiae* postbiotic against *F. oxysporum* infection in fish remain scarce. In the present study, *S. cerevisiae* postbiotic was evaluated specifically for its immunomodulatory activity. The aim was to determine whether dietary supplementation of *S. cerevisiae* could strengthen fish immune responses and thereby increase resistance against experimental *F. oxysporum* infection. This research aimed to fill this knowledge gap by assessing how effectively *S. cerevisiae* postbiotic could reduce susceptibility to *F. oxysporum* infections in fish by using graded levels of dietary *S. cerevisiae* postbiotic supplementation in a 60-day feeding experiment to (1) evaluate growth performance, (2) feed utilization, (3) serum biochemical parameters, (4) the expression of *IGF-1*, *SOD2*, *IL-1β*, and *IL-10* genes in the liver, *IL-1β*, *IL-10*, and *IgM* genes in both spleen and intestine, and (5) disease resistance to *F. oxysporum* in Nile tilapia.

## 2. Materials and Methods

### 2.1. Ethics Statement 

Fish were handled according to the standard protocol approved by the Research Ethics Committee for Animal Use and Care, Faculty of Veterinary Medicine, Assiut University (Code No. 06/2025/0320).

### 2.2. Experimental Fish and Rearing Facilities

Healthy Nile tilapia (*n* = 216) were purchased from a private fish farm in the Assiut Governorate. The fish were brought to the Department of Aquatic Animal Medicine Wet Lab, Faculty of Veterinary Medicine at Assiut University. Fish were kept in 500 L flow-through tanks supplied with dechlorinated water and continuous aeration, fed twice daily with Skretting Tilapia Feed Floating, with analytical values of 30% crude protein and 6% fat (Table 1). Fish were fed at a rate of 3% of body weight and acclimated to the lab conditions for two weeks, according to the protocol for maintaining bioassay fish.

### 2.3. Postbiotic Used in the Current Study

A commercial product was used (DVAQUA^®^ Original XPC ULTRA postbiotic; Diamond V Mills Inc., Cedar Rapids, IA, USA), distributed in Egypt by EgyVet Care (www.egyvetcare.com, accessed on 9 April 2025). The proximate composition according to the manufacturer’s label is listed in Table 2. It is a highly concentrated (8 times more concentrated than original XPC) *S. cerevisiae* fermentation-derived postbiotic. It is a dried product consisting of inactivated yeast cells and the metabolite-rich fermentation medium. It contains bioactive components, including β-glucans, mannan-oligosaccharides, peptides, amino acids, nucleotides, organic acids, vitamins, antioxidants, and other fermentation metabolites [43]. The dosage rates were established by following the manufacturer’s guidelines for the concentrate and by referencing prior peer-reviewed research utilizing Diamond V yeast culture postbiotics. The product is manufactured under a proprietary two-stage fermentation process. It is registered under the Association of American Feed Control Officials (AAFCO) Number: 96.8, International Feed Name (IFN) Number: 7-05-520, and EU Catalogue of Feed Materials: 12.1.12. The product was sourced from a single commercial batch (batch number: U280823), stored under recommended conditions to maintain stability, and no independent compositional analysis was conducted, in line with common practices for proprietary yeast fermentation postbiotics in aquaculture trials [44,45].

### 2.4. Experimental Feed Preparation

Experimental feed was freshly prepared every week. Before the experimental additives were added at their specified levels (1.25, 2.5, or 5 g/kg), the basal diet was ground into a fine powder, sieved to remove lumps, and ground again to ensure a smooth texture. All dry ingredients were then mixed for ten minutes until evenly distributed. Distilled water was added at a rate of 400 mL per kilogram of the mixture, and blending continued for another ten minutes. The moist material was rolled into a sheet 1 mm thick and cut into small pieces suitable for fish feeding. These pieces were dried at room temperature (22–25 °C) using an electric fan until fully dried, and their weight became constant. Once dry, they were placed in sealed plastic bags and stored frozen at −20 °C until needed [29].

### 2.5. Experimental Design

**Figure 1:**
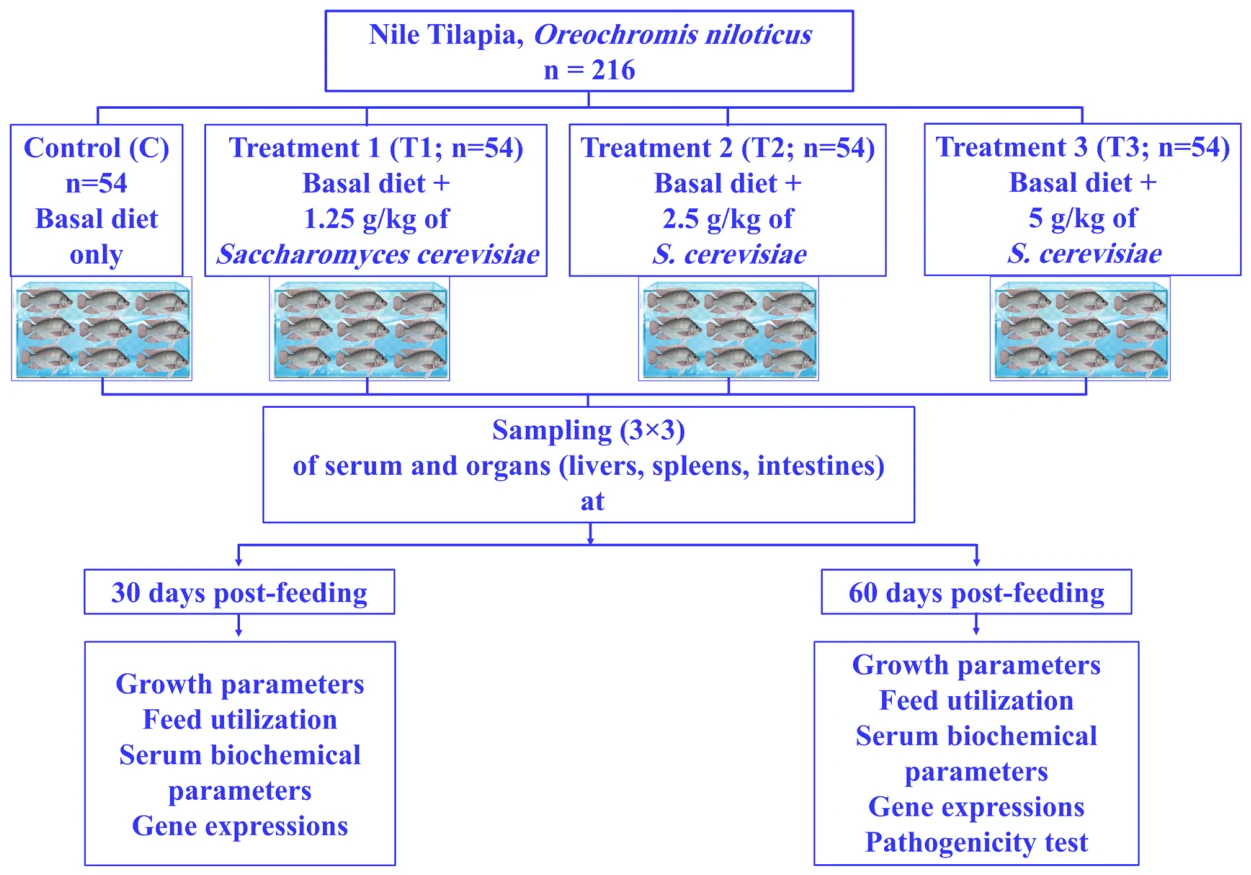
Diagram illustrating the incorporation of various concentrations of *S. cerevisiae* postbiotic into the diet of Nile tilapia for 60 days.

Two hundred and sixteen Nile tilapia, with an average weight of 35.1 ± 0.9 g and an average length of 12.3 ± 0.6 cm, were randomly distributed into four groups. Each group had fifty-four Nile tilapia divided into triplicate groups of eighteen fish. The first group was the control group (C), which was fed the basal diet alone, the second group was treatment one (T1), which was fed the basal diet plus 1.25 g/kg of *S. cerevisiae* postbiotic, the third group was treatment two (T2), which was fed the basal diet plus 2.5 g/kg of *S. cerevisiae* postbiotic, and the fourth group was treatment three (T3), which was fed the basal diet plus 5 g/kg of *S. cerevisiae* postbiotic (Figure 1). For 60 continuous days (from 1 August to 30 September 2025), fish were fed twice a day at intervals of six hours (at 8 am and 2 pm) at a rate of 3% of their body weight. The fish in the tank were weighed and counted collectively every two weeks, and their daily feed intake was recorded and modified based on their weight. The water temperature was adjusted to 25 ± 0.6 °C using a thermo-control aquarium heater (Seastar Aquarium Product Co., LTD, Pollachi, India) and monitored twice daily with an aquarium thermometer (Goxawee). The pH level was measured as 7.2–7.5 with a portable pH meter, and the dissolved oxygen level measured with a portable DO meter was maintained at 6.0–7.0 mg/L. The photoperiod was maintained at a light/dark cycle of 12:12 h. Survival rates were recorded throughout the feeding period (60 days). All experimental fish remained in their original tanks throughout the entire study period (from the start of the feeding trial until the end of the post-challenge observation). No fish were transferred between tanks; thus, handling stress related to transfer was avoided.

#### 2.5.1. Feeding Efficiency and Growth Performance

On the first day of the experiment, each fish’s initial body weight in grams (g; IBW) was recorded. To evaluate the impact of the tested meals on the fish’s health, final body weight (FBW) was measured for each group at 30 and 60 days after the feeding experiment began. For growth parameters, all fish in each replicate were measured and included in the statistical analysis. Fish were fasted for one day before body weight assessment. Furthermore, feeding efficiency and growth performance were computed using the following formulas described by Mohamed, Mahmoud, El Sherry, Abdullah, Bayoumi, Wahman, Mahmoud, Farrag and Abdallah [5]:

Weight gain (WG) = final body weight (g) − initial body weight (g).

Weight gain percentage (%WG) = 100 × ((final body weight (g) − initial body weight (g))/initial body weight (g)).

Feed intake (FI) = total feed consumed by fish during the feeding period (g)/total number of fish.

Feed conversion ratio (FCR) = feed intake (g)/weight gain (g).

Growth rate (GR) = weight gain (g)/duration of the feeding period in days (d).

Specific growth rate (SGR) = 100 × ((ln(final body weight) − ln(initial body weight))/duration of the feeding period in days (d)), where Ln = the natural logarithm.

Survival rate (%) = Number of fish harvested/Number of fish stocked × 100.

#### 2.5.2. Serum and Organ Sampling 

At 30 and 60 days after the start of the feeding experiment, nine (3 × 3) fish were randomly caught from each group. A total of 216 Nile tilapia were randomly distributed into 12 tanks (18 fish per tank). Four dietary treatments were assigned to three replicate tanks each (*n* = 3 tanks per treatment). The tank was considered the experimental unit for all growth performance and feed utilization parameters. Although individual fish within each tank were measured, statistical analyses were performed on tank means. Individual fish were treated as subsamples within each tank. Following fish euthanasia with 60 mg of eugenol (clove oil) per liter of fish tank water [46], blood samples were collected from the caudal blood vessels of Nile tilapia without the use of an anticoagulant. After being centrifuged at 1000× *g* for 10 min, the serum was collected and stored at −80 °C. For gene expression analysis, nine fish were randomly sampled from each dietary treatment at each time point (days 30 and 60). The fish were divided into three groups of three individuals. Within each group, the livers of the three fish were pooled to constitute one biological sample; the spleens of the same three fish were similarly pooled to form one spleen sample; and the intestines were pooled to form one intestine sample. This procedure was repeated for all groups, resulting in three independent biological replicates (*n* = 3) for each organ per treatment and sampling time. Each biological pool was immediately preserved in RNA*later* (Applied Biotechnology, Ismailia, Egypt) and then stored at −80 °C until RNA extraction. On the sampling days, fish were not fed.

#### 2.5.3. Measurement of Serum Biochemical Parameters

Total protein (TP), albumin, globulin, albumin/globulin ratio (A/G), aspartate aminotransferase (AST), creatinine, and glucose were measured using biochemical spectrum diagnostic kits (MDSS GmbH, Germany) in accordance with the manufacturer’s instructions using a spectrophotometer (JENWAY 6315, UK). On the other hand, the total serum albumin was subtracted from total serum protein to determine the total serum globulin.

#### 2.5.4. RNA Extraction and Reverse Transcription

Following the manufacturer’s instructions, total RNA was extracted from all studied organs (50 mg/sample) using the Total and Micro RNA small extraction kit (Applied Biotechnology, Egypt). A nanophotometer (Implen GmbH, Germany) was used to measure the concentration and purity of the RNA. Following the manufacturer’s instructions, a 30 μL quantity of cDNA was produced from each sample using total RNA (1.5 μg) and the RevertAid cDNA synthesis kit (Thermo Scientific, USA).

#### 2.5.5. Quantitative Real-Time PCR (RT-qPCR) and Data Analysis

The QuantStudio^TM^ real-time qPCR detection apparatus (Applied Biosystems, USA) and the Maxima SYBR Green qPCR kit (Thermo, USA) were used for RT-qPCR. The primer set (Macrogen, Seoul, Republic of Korea) was used to determine the expression of *Insulin-like Growth Factor-1 (IGF-1)*, *Superoxide Dismutase 2* (*SOD2*), *Interleukin-1β* (*IL-1β*), *Interleukin-10* (*IL-10*), and immunoglobulin (*IgM*) genes that were previously validated [47,48,49,50,51], and each gene was analyzed in technical triplicate for each biological sample. The cycling profile comprised an initial activation at 95 °C for 10 min, followed by 40 cycles of 95 °C for 30 s, 60 °C for 1 min (annealing), and 72 °C for 1 min (extension). The resulting melting curve analysis confirmed the identification and specificity of the PCR products. The housekeeping gene expression (internal control for cDNA normalization) was the mean of tilapia *β-actin* and *elongation factor* (*EF1α*) gene expression (Table 3), as previously established [5,52,53]. The fold change was calculated using the delta-delta Ct (2^−(ΔΔCt)^) technique according to Livak and Schmittgen [54], and the data were then statistically analyzed.

##### Heatmap and Hierarchical Cluster Analysis with Heatmap Visualization of the Studied Genes

The heatmap was created using GraphPad Prism^®^ 10 Software (version 10.5.0) and a magma colormap. Yellow denotes higher gene expression, while black implies lower gene expression. The color scale shows the color values that correlate to the mean fold change. The core normalization approach is to apply the Z-score (column) to scale data to a common range for comparison, which centers the data around a mean of 0, with a standard deviation of 1. Additionally, a heatmap with a row-clustering dendrogram was created using the expression function in HeatMapper at https://heatmapper.ca/expression/ (accessed on 22 January 2026). Furthermore, a unique custom color scheme was employed, with blue being used for lower expression and yellow for higher expression. Hierarchical clustering was performed using Euclidean distance with average linkage. The data were standardized by column-wise Z-score normalization before clustering. Clustering was performed on rows representing gene expression × treatment combinations, while columns represented the sampling times (30 and 60 days).

#### 2.5.6. Pathogenicity Test (Challenge)

*Fusarium oxysporum* (PZ346033), which was isolated from Nile tilapia mass mortalities in our laboratory, was used in the experimental infection. The pathogen was cultured on potato dextrose agar (PDA) to which 250 µg/mL of chloramphenicol was added [39]. Plates were incubated in the dark at 25 °C for 7 days. Then, the cells were harvested in 20 mL of sterile phosphate-buffered saline (PBS, pH 7.2). The concentration of the conidial cells was adjusted using a hemocytometer [55]. Before the main infection trial, a preliminary study was conducted to establish the median lethal concentration (LC_50_) of the pathogen. Healthy Nile tilapia originating from the same stock were immersed in serial tenfold dilutions of *F. oxysporum* conidial cells. Mortality records were kept daily over 14 days, and the LC_50_ was estimated using the procedure described by Reed and Muench [56].

Thirty fish from each group were immersed for an hour in the tank water containing *F. oxysporum* conidial cells (1 × 10^6^ conidial cells/mL, which equals the LC_50_ that was determined in the preliminary studies in our lab) before being transferred to the corresponding 500 L aquaria containing aerated chlorine-free water. The remaining fish in each group were allocated to sham-control and absolute unchallenged control subgroups to distinguish potential effects associated with handling stress and tank transfer from those attributable to pathogen exposure, and to exclude the influence of environmental factors, respectively. The absolute unchallenged control fish were fed the basal diet, were not exposed to the pathogen, and were maintained under the same experimental conditions. The sham-control fish underwent the same handling procedure as the challenged fish, including transfer to an immersion tank containing pathogen-free water for 1 h, corresponding to the experimental challenge period, after which they were returned to their original tank. The experimental infection was conducted under static conditions in triplicate with 10 fish per replicate. Challenged fish were fed the experimental diet supplemented with graded levels of *S. cerevisiae* postbiotic, monitored twice daily, and dead fish were removed at each observation. Clinical signs and mortality in each group were recorded daily for the next 15 days. Survival percentages were calculated at the end of the experiment. The fungus was reisolated from the infected challenged individuals to fulfill Koch´s postulates. The re-isolation was performed using the identical protocol as the primary isolation. Briefly, tissue samples were aseptically excised, surface-sterilized with 70% ethanol for 30 s, and rinsed twice in sterile phosphate-buffered saline (PBS). The tissues were then homogenized in sterile PBS and plated onto PDA. Plates were incubated at 25 °C for 7 days until visible fungal hyphae emerged. The periphery of the fungal growth was subcultured onto fresh agar plates to obtain pure cultures for morphological characterization.

#### 2.5.7. Statistical Analysis

Feeding efficiency, growth performance, serum biochemical parameters, relative gene expression of *IGF-1*, *SOD2*, *IL-1β*, *IL-10*, and *IgM*, as well as the pathogenicity test data were expressed as means ± SEM. Group differences were analyzed using two-way analysis of variance (ANOVA), followed by Benjamini and Hochberg’s multiple comparisons by controlling the False Discovery Rate (FDR) using the original FDR method, at *p* ≤ 0.05. Before ANOVA, data normality was assessed using the Shapiro–Wilk test, and homogeneity of variance was evaluated. The correlation heatmap was generated using Pearson’s correlation. The Kaplan–Meier method followed by Fisher’s exact test was used to analyze survival percentage following the experimental challenge with *F. oxysporum*, and the log-rank (Mantel-Cox) test was used to evaluate differences between survival curves. Additionally, a dose-dependent association between treatment groups was assessed using a log-rank test for trend. For growth performance parameters and feed efficiency, the tank was considered the experimental unit (*n* = 3 tanks per dietary treatment). Although all fish within each tank were measured, statistical analyses were performed on tank means. Data were analyzed using a two-way analysis of variance (ANOVA) with dietary treatment, sampling time, and their interaction as fixed factors. Individual fish within each tank were treated as subsamples. All analyses were conducted using GraphPad Prism^®^ 10 (version 10.5.0). For each data analysis, both *F* and *p*-values were calculated. 

## 3. Results

### 3.1. Growth Performance and Feeding Efficiency

At 30 and 60 days after the start of the feeding experiment, with a 100% survival rate in all tested groups, the growth performance and feeding efficiency of Nile tilapia were evaluated and summarized in Table 4, showing that FBW and WG increased with both higher postbiotic doses and longer feeding periods. On day 30, both treated groups (T2 and T3) showed significantly higher FBW and WG than the control group, with no significant difference between them. Also, on day 60, the control and T1 groups remained significantly lower than both treated groups (T2 and T3), with no significant difference between T2 and T3 or T1 and control. At both 30 and 60 days post-feeding, T1 had intermediate averages with no significant difference from the control or T2 and T3 groups. WG% increased with both higher postbiotic doses and a longer feeding duration. On day 30, both treated groups (T2 and T3) showed significantly higher WG% than the control and T1 groups, while T3 recorded the highest significant values. On day 60, the control group remained significantly lower than all treated groups (T1, T2, and T3). Among the treatments, T2 values were significantly greater than those of T1, while T3 recorded the highest values, significantly exceeding both T1 and T2. Notably, the control group values on day 60 were comparable to those of T3 on day 30 across all three growth parameters (FBW, WG, and WG%), indicating that the highest dose after 30 days produced growth performance equivalent to that of the non-supplemented group after 60 days of feeding.

Feed intake increased with higher postbiotic doses and a longer feeding period, with T3 recording the only significant increase in feed intake on day 30 compared to the control group. On day 60, the control group recorded significantly lower feed intake than T2 and T3. Notably, there was no significant difference between the control group on day 60 and both T2 and T3 on day 30. FCR decreased with both higher postbiotic dose and a longer feeding period. On day 30, both treated groups (T2 and T3) showed significantly lower FCR than the control group, with no significant difference between them. Furthermore, on day 60, all three treated groups showed no significant difference compared to the control group. Interestingly, the FCR for T2 and T3 on day 30 was similar to that of all groups on day 60.

Both GR and SGR increased with higher postbiotic dose and a longer feeding period; on days 30 and 60, both treated groups (T2 and T3) showed significantly higher GR and SGR than the control group, with no significant difference between them. Interestingly, the GR and SGR for the highest treatment (T3) on day 30 reached levels like those of the control group on day 60.

### 3.2. Serum Biochemical Parameters

Serum biochemical parameter findings are presented in Table 5. The levels of TP, ALB, and GLOB increased as the dosage and feeding period increased. On day 30, TP was significantly higher in the T2 and T3 groups compared with the control and T1 groups, with no significant difference reported between T1 and the control. Additionally, on day 60, total protein continued to rise, with serum TP being significantly higher in all postbiotic-supplemented groups compared to the control, with the highest recorded value in the T3 group, followed by T2 and T1, while the control group showed the lowest reported value, with no significant difference recorded between T1 and T2. Furthermore, T2 and T3 were statistically comparable.

Serum albumin showed only limited changes in response to postbiotic supplementation, with the control, T1, and T2 groups exhibiting similar albumin levels on day 30. T3 was significantly higher than the control and T1 but did not differ significantly from T2. Furthermore, on day 60, the same pattern was observed: control, T1, and T2 remained similar to each other, while T3 was significantly higher than the control and T1, with no significant difference from T2. Notably, the albumin concentrations of the control, T1, and T2 groups were comparable between days 30 and 60.

Serum globulin levels increased with higher postbiotic dose, while the effect of time was more limited. On day 30, T1 showed significantly higher globulin than the control. Both T2 and T3 were significantly higher than T1 and the control, with no significant difference between T2 and T3. On day 60, the same pattern was observed: T1 remained significantly higher than the control, while T2 and T3 were significantly higher than both T1 and the control, and did not differ from each other. Cross-time comparisons revealed that the globulin concentration of T1 on day 30 was similar to both T1 and the control on day 60. In addition, the levels recorded for T2 and T3 on day 30 were comparable to those of T1 and T2 on day 60.

The A/G ratio generally decreased with higher postbiotic doses. On day 30, the control and T1 groups had similar A/G ratios. T1, T2, and T3 did not differ significantly from each other, while both T2 and T3 showed significantly lower ratios than the control. On day 60, T1 was significantly lower than the control. T2 was significantly lower than both the control and T1. T3 was also significantly lower than the control and T1 but did not differ from T2. Cross-time comparisons showed that the A/G ratios of the control and T1 on day 30 were similar to the control on day 60. In addition, the values of T1, T2, and T3 on day 30 were comparable to T1 on day 60. Additionally, T3 on day 60 recorded the lowest A/G ratio among all groups and time points and was similar to T2 on day 60. Finally, the A/G ratio in T3 on day 60 did not significantly differ from its value on day 30.

Furthermore, the AST values showed no significant differences (*p* > 0.05) across all parameters. Moreover, serum creatinine levels were significantly reduced by postbiotic supplementation, with little effect of feeding period or inclusion level. Serum creatinine levels were significantly (*p* ≤ 0.05) lower than control levels in all postbiotic-supplemented groups at 30 and 60 days of feeding. Interestingly, there were no significant differences in creatinine levels between all three treated groups at both checkpoints.

Serum glucose concentration decreased with dietary postbiotic supplementation. On day 30, all treated groups (T1, T2, and T3) showed significantly lower glucose levels than the control. A clear dose-dependent reduction was observed, with T3 recording the lowest value, followed by T2, then T1. On day 60, all treated groups remained significantly lower than the control. T3 was significantly lower than both T1 and T2, while no significant difference was found between T1 and T2. Comparison between the two time points showed that glucose levels of the control group were similar on days 30 and 60. T1 values also remained similar between the two sampling times. In addition, T2 on day 30 was comparable to T1 and T2 on day 60, while T3 maintained similar concentrations on both days 30 and 60.

### 3.3. Studied Gene Expression Profiles, Heat Map Visualization and Hierarchical Cluster Analysis

#### 3.3.1. In the Liver

**Figure 2:**
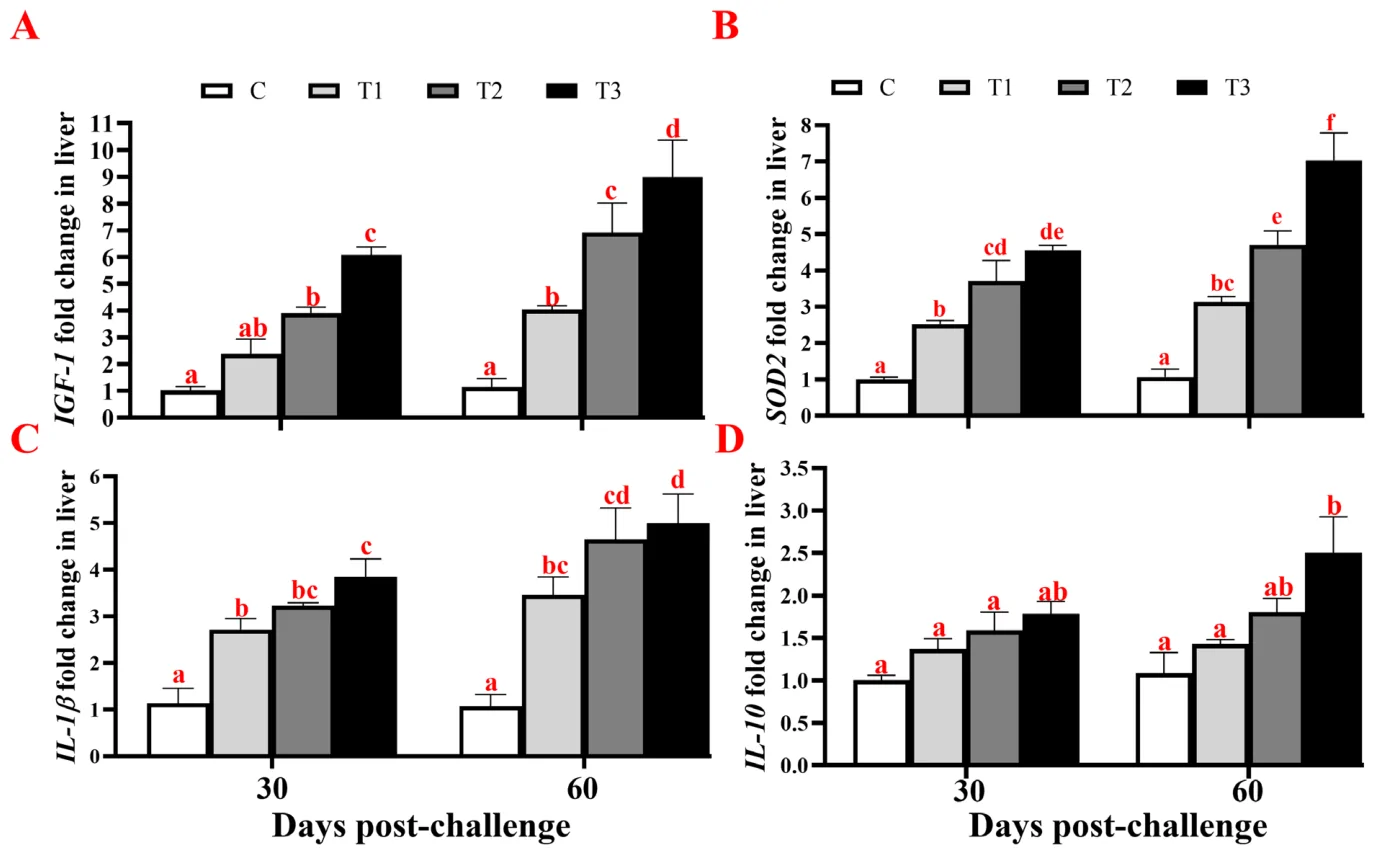
Expression profiles of the studied genes ((**A**): *IGF-1*, (**B**): *SOD2*, (**C**): *IL-1β*, and (**D**): *IL-10*) in the liver of Nile tilapia at 30 and 60 days after the start of the feeding experiment with varying concentrations of *Saccharomyces cerevisiae* postbiotic. The data are expressed as means ± SEM. Different lowercase letters indicate significant differences at *p* < 0.05.

The expression of the *IGF-1* gene (Figure 2A), with a 95% CI of the difference of −3.800 to −0.04443, was significantly upregulated among the treated groups with varying *S. cerevisiae* postbiotic concentrations (*F* (3, 9) = 55.76, *p* < 0.0001) and at different checkpoints (30 and 60 days post-feeding; *F* (1, 3) = 10.61, *p* = 0.0472); however, no significant time × dose interaction was observed (*F* (3, 9) = 3.304, *p* = 0.0714). The expression of the *SOD2* gene (Figure 2B), with a 95% CI of the difference of −1.901 to −0.1767, was significantly upregulated among the tested groups with varying *S. cerevisiae* postbiotic concentrations (*F* (3, 9) = 45.21, *p* < 0.0001) and at different checkpoints (30 and 60 days post-feeding; *F* (1, 3) = 14.70, *p* = 0.0313), and by the interaction between times and different concentrations (*F* (3, 9) = 8.007, *p* = 0.0066). Both the *IGF-1* and *SOD2* genes were upregulated with increases in *S. cerevisiae* postbiotic concentrations and feeding period. The highest transcriptional expression of both *IGF-1* and *SOD2* genes was observed at a 5 g/kg postbiotic inclusion level on day 60. Additionally, the expression of *IL-1β*, a proinflammatory cytokine, and *IL-10*, an anti-inflammatory cytokine, was significantly upregulated, with the *IL-1β* gene expression (Figure 2C), with a 95% CI of the difference of −1.419 to −0.2129, being significantly upregulated in all postbiotic-supplemented groups (*F* (3, 24) = 25.11, *p* < 0.0001) and at both checkpoints (*F* (1, 24) = 7.798, *p* = 0.0101). However, *IL-1β* gene expression had no significant time × dose interaction (*F* (3, 24) = 1.230, *p* = 0.3206). The expression of the *IL-10* gene, with a 95% CI of the difference of −0.7411 to 0.2061, was significantly upregulated only with an increase in the concentration of the administered *S. cerevisiae* postbiotic (*F* (3, 9) = 9.78, *p* = 0.0034), with the highest *IL-10* gene expression being noticed in the 60-day T3; however, there was no significant effect of feeding period (*F* (1, 3) = 3.231, *p* = 0.1701), or time × dose interaction (*F* (3, 9) = 1.066, *p* = 0.4108; Figure 2D).

The highest gene expression in the generated heat map is denoted by yellow, which was observed only in the T3 *IGF-1* gene at 60 days post-feeding with 5.0 g/kg *S. cerevisiae* postbiotic. However, the lowest gene expression, denoted in black, was observed for the *IL-10* gene in T1 and T2 at 30 and 60 days of feeding, as well as in T3 on 30 days of feeding (Supplementary Figure S1).

The similarities and differences in co-expression between genes at the two checkpoints, 30 and 60 days of feeding, were displayed using a heatmap with a row clustering dendrogram. It revealed that the yellow coloration, which indicates the highest expression values, was observed for the expression values of the *IGF-1* gene for T2 and T3 and the *SOD2* gene for T3, which were grouped in a single main branch. Nevertheless, the second main branch was subdivided to include the gene expression for the *IL-10* gene for T1, T2, and T3, which were separated into one major sub-branch, while the second sub-branch was divided to enclose the expression values of the *IL-1β*, *SOD2*, and *IGF-1* genes for T1 in one branch, as well as the expression values of the *SOD2* gene for T2 and the *IL-1β* gene for T2 and T3 in the other sub-branch (Supplementary Figure S2). 

#### 3.3.2. In the Spleen

**Figure 3:**
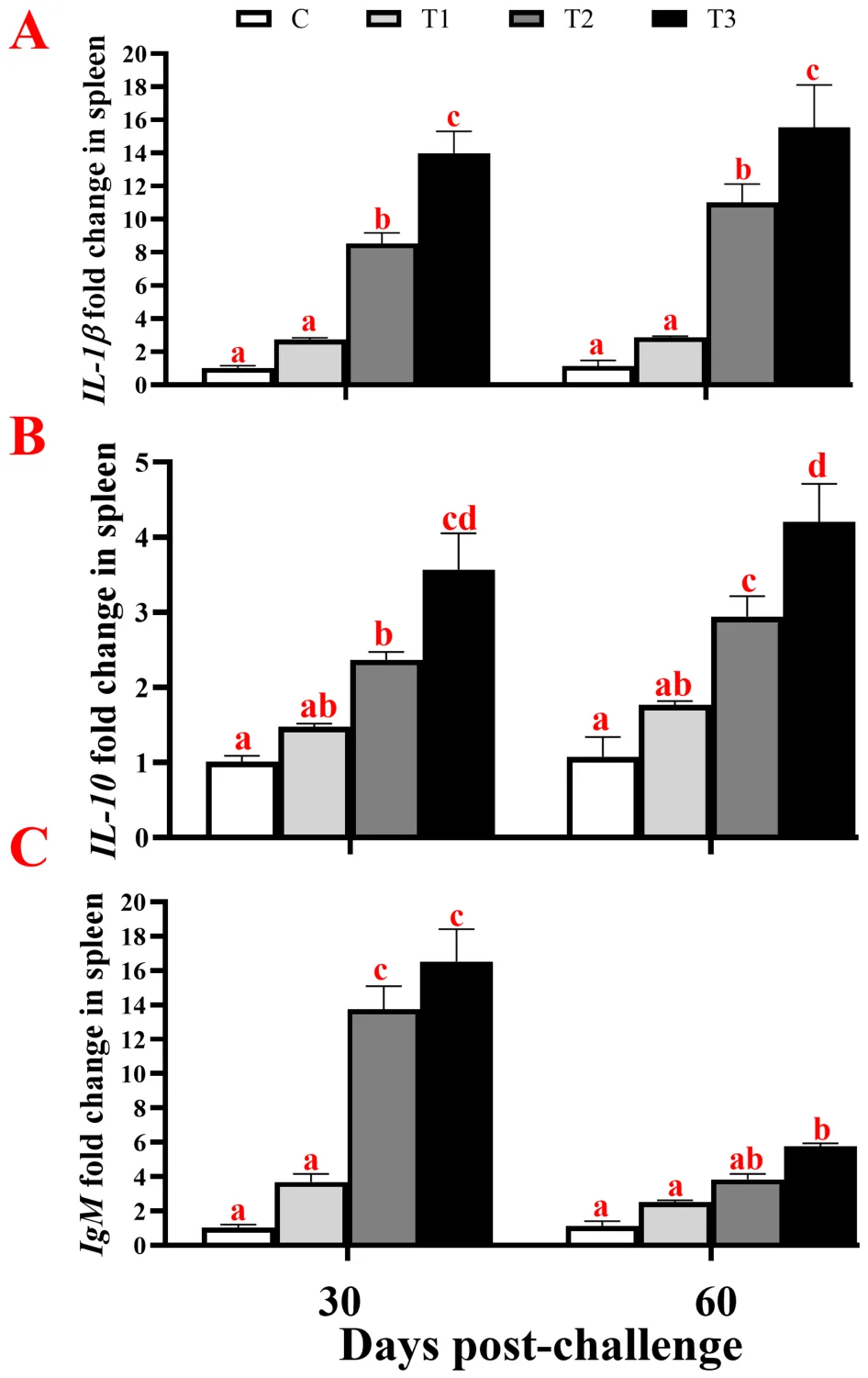
Expression profiles of the studied genes ((**A**): *IL-1β*, (**B**): *IL-10*, and (**C**): *IgM*) in the spleen of Nile tilapia at 30 and 60 days after the start of the feeding experiment with varying concentrations of *Saccharomyces cerevisiae* postbiotic. The data are expressed as means ± SEM. Different lowercase letters indicate significant differences at *p* < 0.05.

The expression of the *IL-1β* gene, with a 95% CI of difference equal to −3.283 to 1.152, was significantly upregulated only when *S. cerevisiae* postbiotic was added to the fish feed compared with the control group. *S. cerevisiae* postbiotic levels (*F* (3, 9) = 48.18, *p* < 0.0001) were positively correlated with *IL-1β*, which was significantly upregulated in all groups, with the same statistical pattern observed in the corresponding treatments at both checkpoints; however, the feeding period (*F* (1, 3) = 2.34, *p* = 0.2236) had no significant effect on *IL-1β* gene expression. When comparing the expression of the three treated groups at both 30 and 60 days of feeding, no significant time × dose interaction was observed (*F* (3, 9) = 0.8617, *p* = 0.4954, Figure 3A). The expression of *IL-10*, with a 95% CI of difference equal to −1.130 to 0.3453, was significantly upregulated only with increasing doses of *S. cerevisiae* postbiotic (*F* (3, 9) = 42.18, *p* < 0.0001) in all groups; however, the feeding period had no significant effect on its expression (*F* (1, 3) = 2.865, *p* = 0.1891). When comparing the expression of the three treated groups at both 30 and 60 days of feeding, no significant time × dose interaction was observed (*F* (3, 9) = 0.4672, *p* = 0.7124, Figure 3B). In contrast, the *IgM* gene, with a 95% CI of difference equal to 3.621 to 7.264, showed significant upregulation with increasing feeding period in all groups at both 30 and 60 days (*F* (1, 3) = 90.40, *p* = 0.0025), and with the use of different doses of *S. cerevisiae* postbiotic (*F* (3, 9) = 55.71, *p* < 0.0001) compared with the control. When comparing the expression of the three treated groups at both 30 and 60 days of feeding, a significant time × dose interaction was noticed (*F* (3, 9) = 24.67, *p* = 0.0001, Figure 3C).

The highest gene expression in the created heatmap is denoted by yellow color, which was only observed in T3, which was fed 5.0 g *S. cerevisiae* postbiotic/kg of fish feed for 30 days in the case of the *IgM* gene level and for 60 days in *IL-1β* gene expression. However, the lowest gene expression, denoted by black color, was observed for the *IL-1β* and *IL-10* genes in T1 at both 30 and 60 days of feeding, and only in T1 at 60 days for the *IgM* gene (Supplementary Figure S3).

The similarities and differences in co-expression between genes at the two checkpoints, 30 and 60 days of feeding, were displayed using a heatmap with a row clustering dendrogram. It revealed that the blue coloration, which indicates the lowest expression values, was observed for the *IL-10* gene for T1, T2, and T3, as well as the *IL-1β* and *IgM* genes for T1, which were grouped in a single main branch. Nevertheless, the second main branch was subdivided to include the gene expression for the *IL-1β* gene for T2 and T3, which was separated into one major sub-branch, and the *IgM* genes for T2 and T3 were clustered together in the second branch (Supplementary Figure S4).

#### 3.3.3. In the Intestine

**Figure 4:**
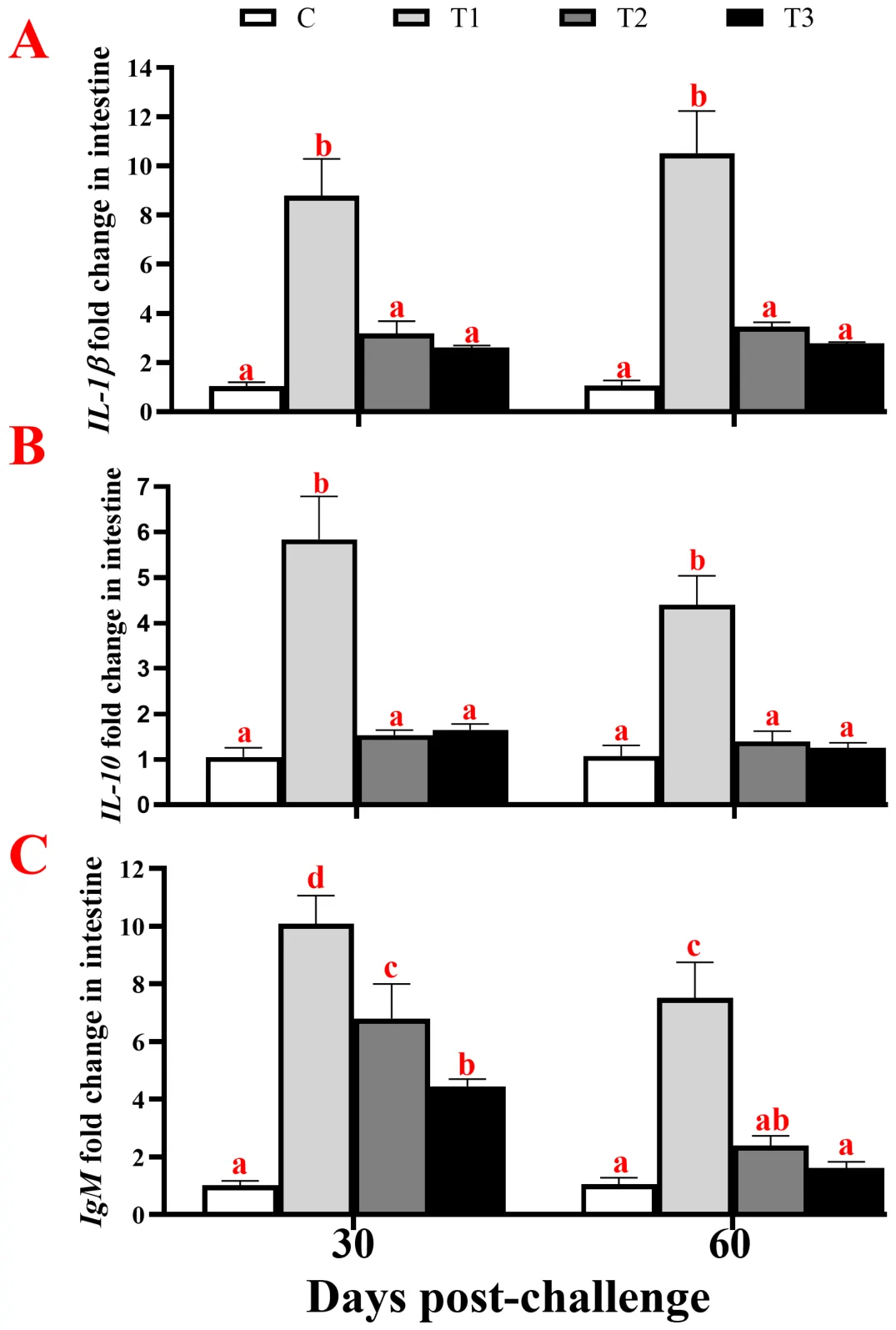
Expression profiles of the studied genes ((**A**): *IL-1β*, (**B**): *IL-10*, and (**C**): *IgM*) in the intestine of Nile tilapia at 30 and 60 days after the start of the feeding experiment with varying concentrations of *Saccharomyces cerevisiae* postbiotic. The data are expressed as means ± SEM. Different lowercase letters indicate significant differences at *p* < 0.05.

Adding *S. cerevisiae* postbiotic to the Nile Tilapia diet resulted in a significant upregulation of the expression of *IL-1β*, *IL-10*, and *IgM* compared to the control group, whereas the *IL-1β* gene expression, with a 95% CI of difference equal to −1.759 to 0.6680, did not differ significantly between 30 and 60 days (*F* (1, 24) = 0.8611, *p* = 0.3627). However, it differed significantly among the treated groups when using different doses of *S. cerevisiae* postbiotic (*F* (3, 24) = 41.19, *p* < 0.0001). Additionally, no significant differences were observed in *IL-1β* gene expression when comparing each treatment group at 30 days and the corresponding treatment group at 60 days of feeding (time x dose interaction; *F* (3, 24) = 0.4474, *p* = 0.7213; Figure 4A). There was a significant upregulation of the *IL-10* gene, with a 95% CI of difference equal to −0.09234 to 1.069, in T1 only at 30 and 60 days post-feeding using different doses of *S. cerevisiae* postbiotic (*F* (3, 9) = 23.80, *p* = 0.0001) compared to the control group. However, no significant effect of either the feeding period (*F* (1, 3) = 7.163, *p* = 0.0753) or the time × dose interaction was observed (*F* (3, 9) = 3.275, *p* = 0.0728; Figure 4B). The expression of the *IgM* gene, with a 95% CI of difference equal to 1.386 to 3.500, was significantly upregulated in all treated groups at both 30 and 60 days post-feeding (*F* (1, 24) = 22.77, *p* < 0.0001) using different doses of *S. cerevisiae* postbiotic (*F* (3, 24) = 41.50, *p* < 0.0001) compared to the control group. When comparing the expression of the three treated groups at both 30 and 60 days of feeding, it was noticed that the *IgM* gene expression was not significantly downregulated (time and dose interaction; *F* (3, 24) = 3.225, *p* = 0.0404) in all treated groups at 60 days compared to the 30-day treated groups (Figure 4C).

The highest gene expression in the created heatmap (yellow-colored) was observed in T1 at 60 days for the *IL-1β* gene level and at 30 days for *IgM* gene expression. However, the lowest gene expression (black-colored) was observed in the case of the *IL-1β* gene level in T3 at both 30 and 60 days post-feeding, in both T2 and T3 at both 30 and 60 days for *IL-10* gene expression, and only in T3 at 60 days in *IgM* gene level (Supplementary Figure S5). 

To illustrate similarities and differences in co-expression between genes across the two checkpoints (30 d and 60 d), a heatmap with a row-clustering dendrogram was created. The *IgM* and *IL-1β* genes for T1 were clustered in a single main branch; however, the second main branch was split into two main sub-branches, with the *IL-10* gene for T1 and the *IgM* gene for T2 clustered in the first sub-branch. The second sub-branch was split into two sub-branches; the *IgM* gene for T3 and the *IL-1β* gene for T2 were clustered together in the first sub-branch, and the *IL-10* gene for T2 and T3 and the *IL-1β* gene for T3 were clustered together in the second sub-branch (Supplementary Figure S6).

### 3.4. Heatmap Visualization of Maximum Fold Change of Expression Profiles of the Studied Genes in Different Organs Using Colors

Heatmap visualization of numeric data using colors was performed using GraphPad Prism^®^ 10 Software (version 10.5.0). Color values equal to the mean fold change are represented on a color scale ranging from black for the lowest expression to yellow for the highest expression. In the current investigation, the yellow color represents the highest expression of *IL-1β* in the spleen for the third treatment at 30 and 60 days post-feeding (Supplementary Figure S7). Additionally, for the *IL-10* gene, the yellow color indicates the highest expression in the first treatment at 30 d, followed by 60 days post-feeding, with no significant difference between them in the intestine (Supplementary Figure S8). Additionally, the yellow color indicates the highest expression of *IgM* in the spleen at 30 d for the second (T2; fed 2.5 g/kg *S. cerevisiae* postbiotic) and third (T3; fed 5 g/kg *S. cerevisiae* postbiotic) treatments, with no significant difference between them (Supplementary Figure S9).

### 3.5. Exploratory Correlation Analysis

**Figure 5:**
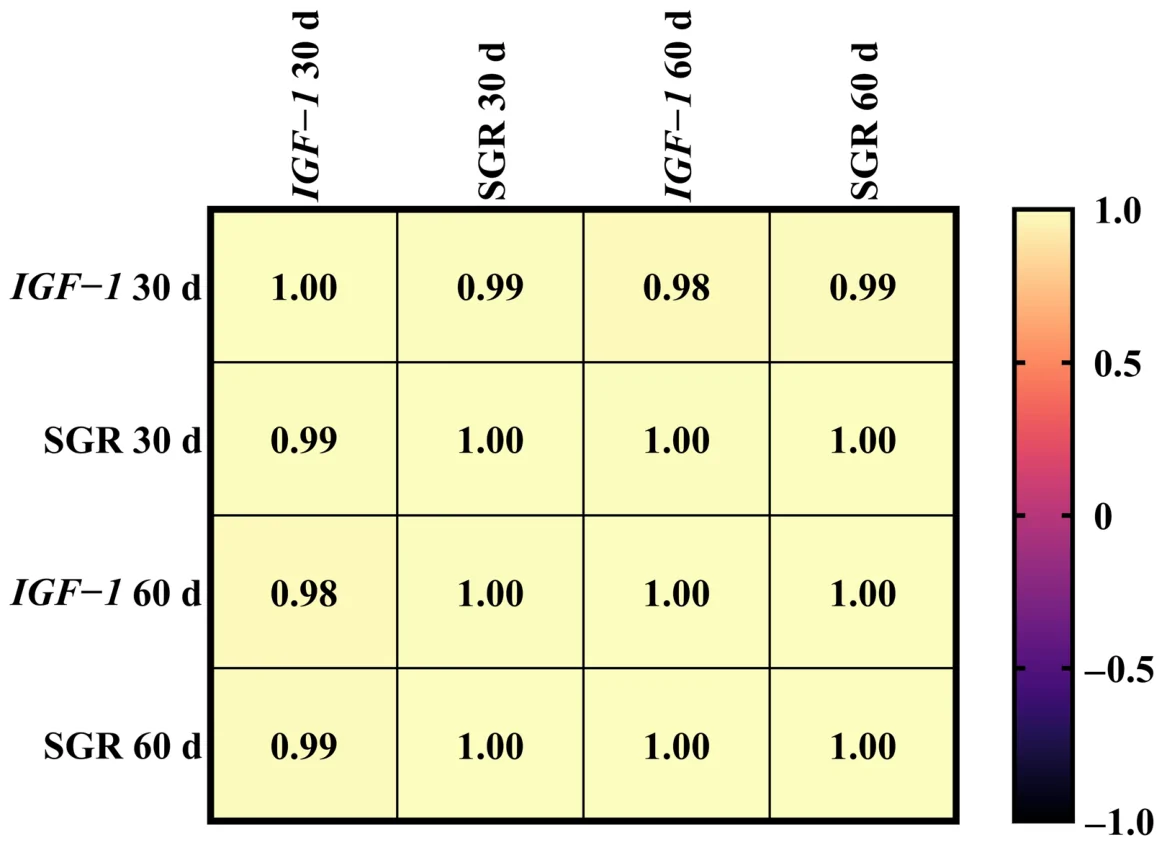
Correlation heatmap analysis between the specific growth rate (SGR) and insulin-like growth factor 1 (*IGF-1*) gene expression in the liver of Nile tilapia (*Oreochromis niloticus*) after the administration of different concentrations of *Saccharomyces cerevisiae* postbiotic for 30 and 60 days following the start of the feeding experiment.

The specific growth rate at both 30 and 60 days was positively correlated with the expression of the *IGF-1* gene, as determined using Pearson’s correlation coefficient (*p* < 0.05; Figure 5).

### 3.6. Pathogenicity Test (Challenge)

**Figure 6:**
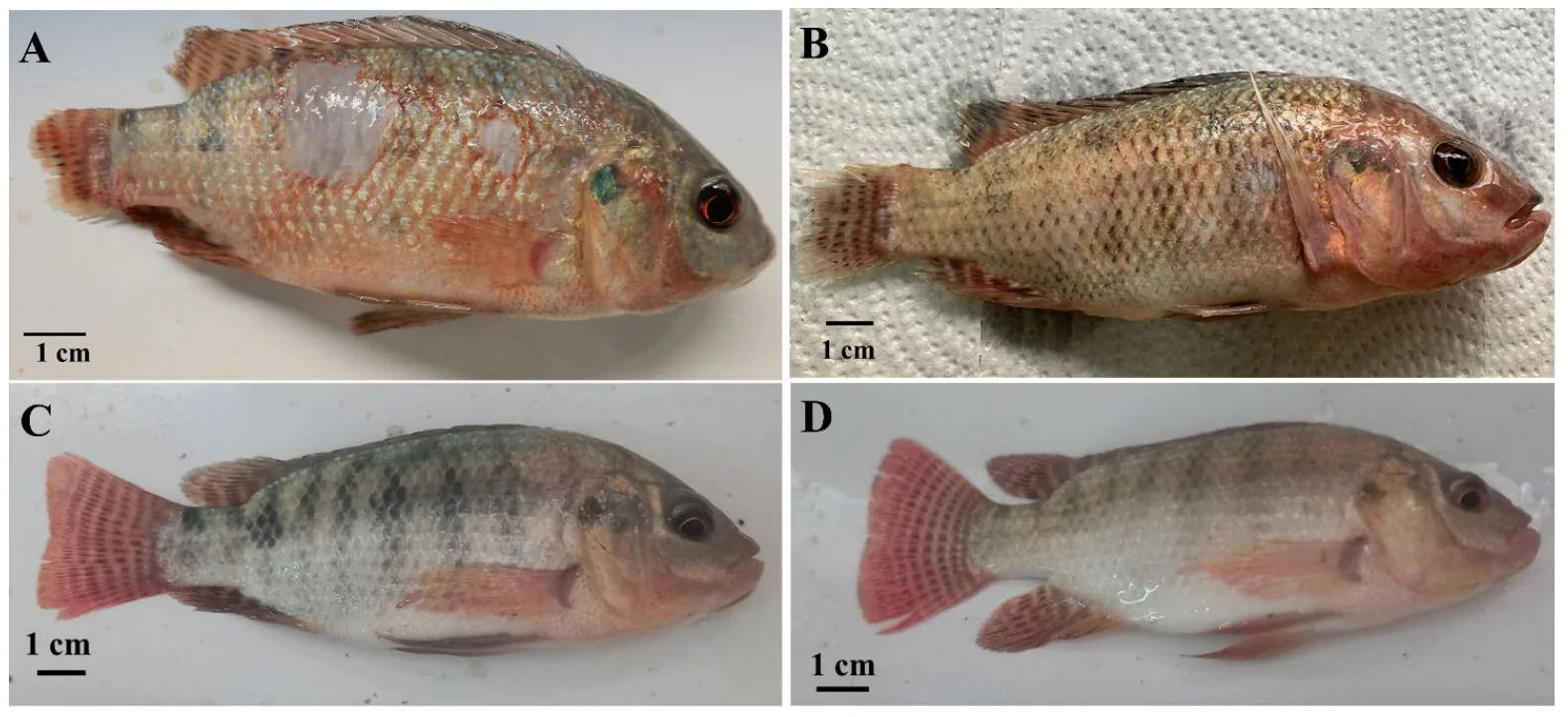
Nile tilapia were administered different concentrations of *Saccharomyces cerevisiae* postbiotic ((**A**): 0 g/kg; (**B**): 1.25 g/kg; (**C**): 2.5 g/kg; and (**D**): 5 g/kg of feed) for 60 days and were challenged with *Fusarium oxysporum* (PZ346033) through immersion for an hour in tank water containing 1 × 10^6^ conidial cells/mL.

**Figure 7:**
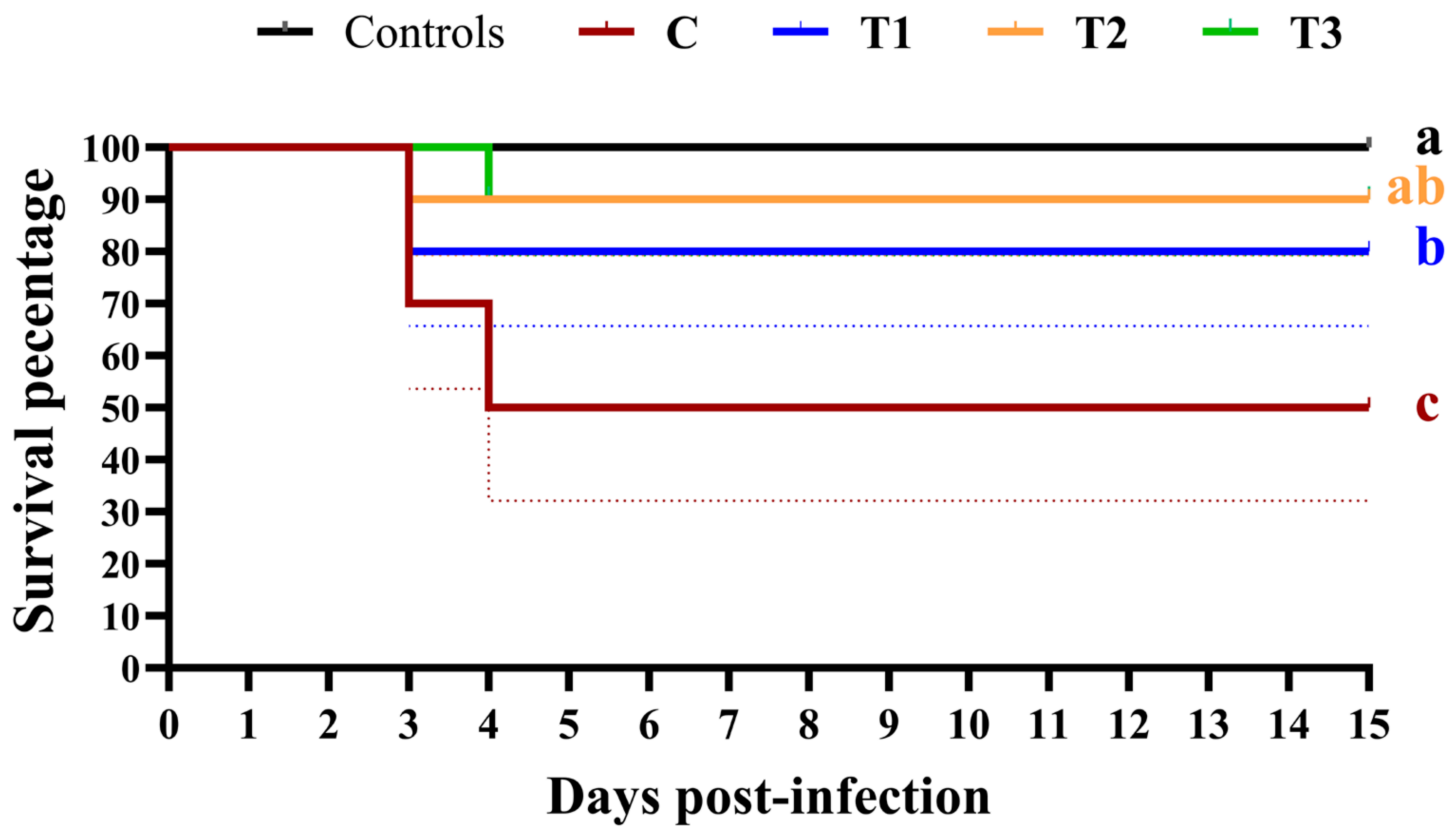
Survival percentage of Nile tilapia fed a commercial ration supplemented with graded levels of *Saccharomyces cerevisiae* postbiotic for 60 days and challenged with *Fusarium oxysporum* (PZ346033). Data are presented as means ± SEM, where solid lines indicate the average, while dashed lines indicate error bars. Different lowercase letters indicate significant differences at *p* < 0.05.

The fish began to die on the third and fourth days post-challenge, exhibiting signs of increased mucus discharge, scale separation, and hemorrhages in various parts of their bodies (Figure 6). The observed clinical signs were more pronounced in the control group compared to all treated groups, with complete sloughing of the skin, frayed fins, and hemorrhages all over their bodies being observed in the control-challenged fish that received no *S. cerevisiae* postbiotic supplementation (Figure 6A); however, small ulcerations with frayed fins and hemorrhages over their bodies were seen in T1 (Figure 6B). In contrast, fish in T2 (Figure 6C) and T3 (Figure 6D) appeared healthy (Figure 6C). *F. oxysporum* was re-isolated on PDA from the internal organs (livers, kidneys, and spleens), as well as the blood of the moribund fish, and was identified morphologically. The survival rates of the experimentally challenged groups varied significantly according to the Kaplan–Meier survival analysis (log-rank Mantel–Cox test: χ^2^ = 30.97, df = 4, *p* < 0.0001). A substantial dose-dependent association between groups was confirmed by the log-rank test for trend (χ^2^ = 24.93, df = 1, *p* < 0.0001), which is consistent with a progressive improvement in survival with increasing *S. cerevisiae* postbiotic supplementation. All fish fed *S. cerevisiae* postbiotic-supplemented treatments showed greater survival rates than those fed the control treatment in a dose-dependent manner, with no significant difference between the second and third treatments (Figure 7). 

## 4. Discussion

### 4.1. Feeding Efficiency and Growth Performance

The next generation of probiotics, known as postbiotics, is distinguished by its inactive nature, with inactivated microbial cells, components of bacterial/yeast cells, and crude by-products derived from microbial metabolism [57]. *S. cerevisiae* postbiotic has many growth factors that could alter the microecological balance of aquatic animals’ digestive tracts [12]. In the current investigation, at 30 and 60 days post-feeding with *S. cerevisiae* postbiotic, higher inclusion levels of 2.5 and 5 g/kg (especially 5g/kg) resulted in significant increases in final body weight, weight gain, weight gain percentage, feed intake, growth rate, and specific growth rate (*p* ≤ 0.05) and decreases in the feed conversion ratio compared to the control group. Adding an appropriate amount of *S. cerevisiae* to aquatic feed can significantly improve the specific growth rate and weight gain of Nile tilapia [58], rainbow trout [59], and rohu [60]. *S. cerevisiae* as a feed additive added in crucian carp, juvenile common carp, and channel catfish diet improved fish growth performance and feed conversion efficiency [12,61]. That may be attributed to its potential influence on growth-related factors and modification of the microecological balance of aquatic animals’ digestive tracts [12]. *S. cerevisiae* postbiotic’s rich nutritional composition, which includes proteins, carbohydrates, nucleic acids, high β-glucans, and mannan oligosaccharides (MOS), is essential for supporting fish development [26]. The commercial fermentation-derived product has a complex composition containing MOS and β-glucan, with MOS used in rainbow trout increasing both weight gain and feed utilization efficiency [62]. Moreover, the inclusion of β-glucan in rohu fingerlings, Nile tilapia, and the diet of pompano fish (*Trachinotus ovatus*) significantly increased feed intake and growth parameters [63,64,65]. That may result from the breakdown of glucan by the digestive glands’ β-glucanase enzyme which promotes the use of more protein for growth (protein-saver effect) [66]. Similarly, a mixture of MOS and β-glucan dietary supplementation improved Nile tilapia’s weight gain and feed conversion ratio [37].

Previous experimental studies have demonstrated variable responses to DVAQUA^®^ (Cedar Rapids, IA, USA) postbiotic supplementation among different aquaculture species. In rainbow trout, dietary supplementation was associated with reduced mortality and, in some trials, improved growth and survival, particularly at higher inclusion levels and early life stages [67,68]. Positive growth responses were also reported in lake trout and brown trout, although the effects varied among strains and species, with limited responses observed in some trout populations [68,69]. In hybrid tilapia, DVAQUA^®^ postbiotic supplementation generally had no significant effects on growth performance, feed intake, feed conversion, or survival under normal rearing conditions; however, it consistently enhanced several nonspecific immune responses, including serum lysozyme activity, complement components C3 and C4, macrophage phagocytosis, and respiratory burst activity [70,71].

In Pacific white shrimp, supplementation, particularly at 0.35%, improved final weight, weight gain, average daily growth, and specific growth rate [45]. In Nile tilapia and channel catfish, DVAQUA^®^ postbiotic had limited effects on growth performance, although lower supplementation levels improved survival in some cases. Nile tilapia showed improved survival following *Streptococcus iniae* challenge [44]. McConaughy rainbow trout is a wild fish strain that is harder to raise in hatcheries and survives better in natural lakes and rivers. Barnes and Durben [67] attributed the improvement in its growth especially in the final stage of rearing, to the nutritional value of *S. cerevisiae*, suggesting that it may colonize the fish’s gut and produce beneficial nutrients, increase palatability, and encourage the fish to eat more. Barnes, Fletcher, Dakota and Durben [69] recommended *S. cerevisiae* postbiotic for salmonids, particularly those hesitant to accept standard formulated feeds.

When β-glucans are administered orally, they are absorbed in the intestine after passing through the digestive system. Nevertheless, the fish intestine does not completely digest and absorb β-glucans. The only orally administered β-glucans that can cross the intestinal wall, enter the bloodstream, and reach targeted organs are those that are smaller than 1 μm [72]. This could be explained by previous studies that indicated a potential relationship between dosage relative to species-specific recommendations and the achievement of significant growth responses. Dalmo and Bøgwald [73] stated that while dietary ß-glucans from *S. cerevisiae* can promote faster growth in some fish under particular circumstances, other experiments have failed to replicate this effect using different ß-glucan preparations. They suggested that the growth-promoting effect appears to depend on many factors, including the dietary inclusion level, feeding period, rearing temperature, and the particular species involved.

The complex composition of the commercial postbiotic used in this study, which, according to the product label, includes various amino acids, may contribute to its beneficial effects on growth performance, protein synthesis, antioxidant defense, immune responses, intestinal health, and feed utilization in fish. Amino acids such as threonine, methionine, lysine, valine, leucine, and proline may be partly related to muscle growth and protein synthesis through the activation of the *GH*/*IGF-1* and PI3K/AKT/TOR signaling pathways, and their dietary supplementation has been widely reported to improve growth performance, feed efficiency, and protein utilization [74,75,76,77,78,79,80]. Arginine may be linked to supporting energy metabolism and muscle protein synthesis through AMPK and TOR activation and may also exert immunomodulatory effects by regulating inflammatory responses and enhancing host defense [81]. Serum biochemical parameters, growth performance, and feed conversion are potentially mediated by isoleucine, whereas histidine was previously reported in many studies to enhance digestive enzyme activities and growth [82].

### 4.2. Serum Biochemical Parameters 

Serum biochemical parameters are widely recognized as indicators of overall fish health, with total protein, globulin, and albumin representing the major protein fractions [83]. The results of the current investigation showed that when the concentration of the administered *S. cerevisiae* postbiotic in the diet increased, total protein, albumin, and globulin levels increased; however, the albumin-to-globulin ratio decreased. It was reported that total protein and albumin were increased with increasing *S. cerevisiae* extract levels in a tilapia diet [84]. That could be explained by the postbiotic’s rich amino acid content, declared by its manufacturer, and the role of these amino acids in the protein synthesis pathway. For example, lysine and methionine may contribute to higher serum total protein levels by activating the TOR signaling pathway (promoting protein synthesis) and reducing protein degradation pathways, thereby improving overall protein status [77,78]. Additionally, the postbiotic used in this study was also declared by its manufacturer to contain nucleotides; increased dietary nucleotide levels might lead to an increase in serum total protein, albumin, and globulin in rainbow trout [85].

Plasma or serum enzyme activity levels of AST may be suggestive of hepatic function and the health status of fish. Therefore, in the clinical diagnosis of hepatic health, AST is a typical activity value for “liver function” testing. An elevation in serum AST enzyme activity indicates liver damage or inflammation induced by environmental pollutants, disease, stress, or nutrient deficiencies [86]. In the current study, AST in all supplemented groups did not significantly differ from the normal control group. 

The serum creatinine levels discovered in all tested groups in the current investigation fell within the reference ranges (0.2–0.8 mg/dL) indicated in earlier research [87], with the *S. cerevisiae* postbiotic-fed groups exhibiting significantly lower serum creatinine levels than the control group fed a basal diet. Lower creatinine levels have been described in the literature as an indicator of good kidney function [88]. Previous findings suggested that the presence of β-Glucan derived from *S. cerevisiae* was reported to promote the release of granulocyte-colony-stimulating factor (G-CSF) from macrophages, endothelial cells, and lymphocytes. The resulting rise in G-CSF expands the pool of circulating hematopoietic stem cells (HSCs), which contributes to the repair of injured renal tubular epithelial cells. This regenerative process improves kidney function and leads to a reduction in serum creatinine concentrations [89]. However, further renal-specific investigations including histopathological analysis are required to clarify the biological significance of this response.

Furthermore, compared to the control in the present study, glucose values significantly decreased with increasing inclusion levels. This could be explained by the effects of β-glucans, as it was reported that dietary β-glucan at 1 g kg^−1^ significantly improved total immunoglobulin levels in striped catfish fingerlings after 30 days of feeding, while simultaneously lowering plasma glucose levels [90]. When these fish were subjected to crowding stress, those previously fed the same dose for three weeks showed significantly lower glucose levels and maintained higher immunoglobulin levels compared with shorter feeding periods. These findings indicate that an optimal dietary inclusion of 1 g β-glucan kg^−1^ can enhance humoral immunity and stress resistance in this species [90]. Along with insulin signaling, the *IGF-1* pathway contributes importantly to preserving glucose homeostasis in metabolic processes [91]. The upregulation of hepatic *IGF-1* gene expression in the current investigation may be attributed to the presence of methionine, arginine, and lysine, which increase the expression of hepatic insulin-like growth factor-1 (*IGF-1*) and serum insulin (INS) levels [76,77,78,81]. *IGF-1* receptor a (igf1ra) maintains sugar metabolic balance. Its absence causes high blood sugar and increased liver sugar storage. In zebrafish lacking the igf1ra−/− receptor, the expression of genes involved in glucose uptake and glycolysis was significantly reduced, while genes associated with gluconeogenesis and glycogen synthesis showed increased expression [91].

### 4.3. Expression Profiles of the Studied Genes

Nutritional status affects the production of hepatic and circulatory *IGF-1* in several fish species [47,92]. The detection of *IGF-1* is becoming more popular as a potential biomarker for piscine growth rate [47,92] because the association between *IGF-1* levels and growth rate is more consistent than that between growth hormone and growth rate [47]. According to the correlation heatmap analysis, hepatic *IGF-1* mRNA expression and the growth outcomes reported in this investigation were positively correlated under different feeding regimes with different concentrations of *S. cerevisiae* postbiotic and different feeding intervals. 

Antioxidant reactions are considered the most essential defensive systems that protect living organisms from adverse environmental conditions. The *SOD2* gene was upregulated at the transcriptional level in the current investigation. The elevated *SOD2* mRNA levels indicate transcriptional regulation of the antioxidant defense system. This may be attributed to the effects of the main components of the *S. cerevisiae* cell wall, β-glucan, and MOS, as a decrease in reactive oxygen metabolites has been shown in red seabream (*Pagrus major*) fed β-glucan at different concentrations compared to the control group [93]. Additionally, β-glucan enabled Nile tilapia to resist *A. hydrophila* by modulating antioxidant capacity [94], and MOS positively improved *SOD* activity in sea cucumbers (*Apostichopus japonicus*) and juvenile Pacific white shrimp [95]. The amino acids in the postbiotic amino acid profile, including cysteine, phenylalanine, and proline, have been documented to contribute to antioxidant protection by modulating *SOD*, *CAT*, *GPx*, and other antioxidant systems, while cysteine may be related to modulating NF-κB-related inflammatory signaling and supporting intestinal barrier integrity and microbiota composition [80,96,97,98,99]. Tryptophan may improve antioxidant status and has been associated with enhanced growth and feed utilization [100]. 

In the current investigation, the hepatic expression of *IL-1β* and *IL-10* genes was significantly increased by *S. cerevisiae* postbiotic supplementation. This is potentially mediated by the presence of β-glucan, which has been shown to contribute significantly to immune modulation in Nile tilapia [101], zebrafish [102], rainbow trout [36], common carp [103], Pacific white shrimp [104], and sea cucumbers [105]. In fish, Toll-like receptors (TLRs) function as sensing receptors, detecting the presence of β-glucans and triggering the production of proinflammatory cytokines such as *IL-1β*, *TNF*, and *IL-8* to modulate the immune response [106]. Additionally, β-glucans can inhibit the excessive activation of the inflammatory response by increasing the transcription of anti-inflammatory cytokines, such as *IL-10* [107].

The expression of *IL-1β* and *IL-10* genes did not differ significantly between T2 and T3 or between the two time points studied. This may be explained by the action of β-glucans in strengthening the fish’s immune system, which also depends on the dosage, frequency, and duration of β-glucan administration [108]. Continuous administration of a β-glucan diet has also been shown to induce the development of tolerance and may result in immune fatigue in koi (*C. carpio koi*) [109]. Dietary supplementation with *S. cerevisiae* postbiotic significantly upregulated the splenic expression of *IL-1β* and *IL-10* genes at both time points. The expression was significantly increased with increasing *S. cerevisiae* postbiotic inclusion levels [110], while intestinal *IL-1β* and *IL-10* gene expression was comparable to that of the control group, except for the lowest dietary *S. cerevisiae* postbiotic concentrations, which showed significantly higher expression levels than those of all groups at both 30 and 60 days post-feeding. The simultaneous upregulation of *IL-1β* and *IL-10* genes in the liver and spleen, together with their downregulation in the intestine, is consistent with a tissue-specific immunomodulatory response. According to previous research, the rich composition of the postbiotic, including β-glucans, may regulate immune response in an organ-dependent manner [111]. When β-glucans were orally administered, the immunomodulatory effect would first be induced in the gut, followed by other lymphoid organs. Hence, the acute inflammation in the gut was reduced via downregulation of cytokine expression, and at the same time, the inflammatory process began to activate in the anterior kidney [108]. The spleen is a highly responsive immunological organ to dietary β-glucans, in addition to the intestine, as the primary regulation of immune-related genes such as *Il-1β*, *IL-10*, and *TGF-β* was shown in the spleens of rainbow trout following β-glucan administration [112]. The elevated expression of proinflammatory mediators in the head kidney and spleen may be due to β-glucans’ activation of the TLR signaling pathway [94]. Since proinflammatory cytokines like *TNF-α* and *IL-1β* are essential for the immune response’s adjustment, several studies have suggested that examining their expression can help predict variations in the immune response [34]. 

In the present study, *IgM* gene expression showed tissue- and time-specific patterns, as the splenic *IgM* gene expression was significantly upregulated in T2 and T3 on day 30, while only T3 remained elevated on day 60, although at a lower absolute level than on day 30. In the intestine, all doses increased *IgM* gene expression on day 30 in a reverse dose-dependent manner and by day 60, only T1 stayed significantly higher than the control. The effectiveness of postbiotic complex content, including β-glucans, in strengthening the fish’s immune system depends on the feeding period and frequency of administration [108]. Low and moderate levels of β-glucan induced upregulation of *IgM* gene expression in Japanese flounder (*Paralichthys olivaceus*) [113]. β-glucans are absorbed in the intestine after passing through the digestive system following oral administration [72]. As immunomodulators, β-glucans function as pathogen-associated molecular patterns (PAMPs) recognized by pattern recognition receptors (PRRs) on immune cells. Dectin-1 is considered the major β-glucan receptor, often working in concert with Toll-like receptor 2 (TLR2) to initiate intracellular signaling. This recognition initiates a cascade of immune responses, including the upregulation of proinflammatory cytokines, enhanced phagocytic activity, and the activation of the complement system [73]. The immunomodulating activity of β-glucan can be attributed to the interaction between beneficial microorganism cells and the intestinal epithelial cells, which can probably improve mucosal immunity in the host gut, thereby tightening the epithelial junctions, producing antimicrobial peptides and mucosal immunoglobulin, modulating inflammatory reaction, and consequently stimulating the immune response. This expression pattern at higher doses may be related to prolonged dietary exposure to high levels of β-glucans, which has previously been shown to induce immune tolerance and potential immune fatigue in koi [109]. Furthermore, dietary β-glucans have dose-dependent effects on juvenile rainbow trout, whereby low doses may modulate immune function and inflammatory responses during bacterial infection, while high doses cause immune exhaustion or feedback regulation, impairing the ability to respond to pathogens [112]. Higher doses of β-glucans will overburden the immune cells, resulting in excessive stimulation that lowers immune cell activity and cause immune exhaustion [108]. 

Selecting the anterior intestine for gene expression analysis of *IL-1β*, *IL-10*, and *IgM* was based on its primary immunological and functional role in teleost fish. The anterior intestine is the initial segment responsible for nutrient absorption and early exposure to dietary antigens, feed additives, commensal microbiota, and potential pathogens after gastric processing [114,115]. Scientists studied the expression of cytokines in rainbow trout during infection with *Aeromonas salmonicida*, and they found that, in the anterior part of the intestine, several immune signals (cytokines, including *IL-1β*, *IL-8*, *TNF-α*, and *IFN-γ*) that trigger inflammation became more active, while no significant changes were recorded in the posterior intestine [114]. Regional specialization along the gut supports the choice of the anterior intestine for the current study, as the anterior portion showed stronger proinflammatory cytokine responses and immune activation compared with the posterior segments. Sampling here thus provides a sensitive and representative measure of early mucosal immune changes relevant to this study’s focus on gut-associated immunity. This practice aligns with established methodologies in teleost immunology, in which anterior intestinal tissue is commonly analyzed for cytokine (*IL-1β*, *IL-10*) and *IgM* gene expression to assess responses to immunostimulants, infections, or dietary interventions [5].

### 4.4. Pathogenicity Test (Challenge)

Fish survival rate is correlated with the activation of nonspecific immunity [37]. In the present study, the observed clinical signs and pathological changes were consistent with those of *F. oxyspoum* [41]. Additionally, fish fed *S. cerevisiae* postbiotic demonstrated higher survival rates in a dose-dependent manner across treatments compared to the control, especially with increasing levels of *S. cerevisiae* postbiotic; this may be due to the complex components of postbiotic including β-glucans, which are responsible for a multitude of actions that protect and modulate the immune system and provide optimum resistance to any potential health assailants because they can directly bind to and activate macrophages and other white blood cells, such as neutrophils and natural killer (NK) cells [116,117]. Postbiotic-supplemented fish feed modulated immunological responses against pathogens, encompassing humoral and cell-mediated responses [118]. Nile tilapia fed *S. cerevisiae* and subsequently challenged with *A. hydrophila* exhibited lower mortality rates compared to supplemented controls [119]. It is important to distinguish between two different modes of action of functional feed additives. Some ingredients induce a direct antimicrobial effect by inhibiting or killing pathogens (bacteria or fungi) through compounds such as organic acids, bacteriocins, or other antimicrobial metabolites. In contrast, other ingredients act as immunostimulants or immunomodulators, enhancing or regulating the host’s own immune system to improve disease resistance. In the present study, *S. cerevisiae* fermentation-derived postbiotic was evaluated for its immunomodulatory activity, not for the antifungal action of the product.

This study has some limitations that should be acknowledged. First, there was an absence of independent compositional analysis of the specific batch of commercial *S. cerevisiae* postbiotic used. Because the product is proprietary, detailed quantitative data on individual bioactive constituents and the precise inactivation method were not available from the manufacturer. Consequently, the observed effects are attributed to the product as a whole rather than to any single component. Future studies incorporating detailed chemical characterization of the additive would allow more precise mechanistic interpretation. Second, this study relied on selected serum and gene expression markers, so broader molecular profiling would provide a more complete understanding of the postbiotic’s mode of action. Third, the experiment was conducted under controlled conditions, and the results may not fully reflect responses under commercial farming environments where stressors are more variable. Fourth, the final experimental diets were not subjected to independent proximate analysis. Although the postbiotic was included at relatively low levels (1.25–5 g/kg), its contribution to the final nutrient composition was not experimentally quantified. Future studies should characterize the proximate composition and energy content of the finished diets to confirm their nutritional equivalence and further strengthen attribution of the observed effects to postbiotic supplementation. Finally, although the data support a positive effect of *S. cerevisiae* postbiotic, further studies comparing this product with other postbiotic or functional feed additives would help clarify its relative effectiveness in Nile tilapia nutrition and health management.

## 5. Conclusions

The current investigation demonstrated that dietary supplementation with 5 g/kg *S. cerevisiae* postbiotic for 60 days significantly improved weight gain, reaching up to 175.6 ± 27.0%, specific growth rate up to 1.5 ± 0.1%, and feed conversion ratio up to 0.8 ± 0.2 compared to the control group. The supplementation could alter serum biochemical parameters (increasing total protein, albumin, and globulin and decreasing glucose levels), consistent with changes in the metabolic and physiological status of Nile tilapia. Additionally, it modulated gene expression in Nile tilapia; however, the magnitude and direction of responses varied according to dose, tissue, and target gene. A significant decrease in the susceptibility of Nile tilapia to *F. oxysporum* infection, accompanied by higher survival rates with less severe clinical signs, highlights favorable immunomodulation. However, to translate these promising laboratory findings into practical applications, in vitro antifungal effects and larger-scale field trials under commercial farming conditions are essential. Such trials should evaluate economic feasibility, consistency of growth and health benefits across different production systems, and potential synergistic effects with other feed additives. Future research addressing these aspects will be critical for establishing *S. cerevisiae* postbiotic as a reliable functional feed ingredient in intensive Nile tilapia aquaculture.

## Figures and Tables

**Table 1:** Analysis of the basal diet ingredients and chemical constituents (dry weight, %).

Ingredients	% In Fish Ration
Fish meal (65%)	9
Soybean meal (46%)	36.85
Corn gluten (60%)	12.2
Yellow corn	19.25
Wheat bran	5.7
Fish oil	6.00
Starch	7.00
Mineral premix (without se)	2.00
Vitamin premix	2.00
Total	100
Proximate composition % dry matter (DM)	%
Crude protein (CP)	30
Crude fiber (CF)	4.8
Ash	8.2
Ether extract (EE)	6.5
* Nitrogen-free extract (NFE)	50.5
Energy	3990 Kcal

* NFE = 100 − (CP% + EE% + CF% + Ash%).

**Table 2:** Proximate composition of DVAQUA^®^ postbiotic.

Analysis	Specification	Analysis	Specification	Analysis	Specification
Crude protein (min)	19.0%	Sodium (Na)	0.9%	Proline	0.94%
Moisture (max)	11%	Sulfur (S)	0.71%	Tyrosine	0.58%
NFE	≥40%	Isoleucine	0.59%	Tryptophan	0.16%
ADF	23.60%	Valine	0.76%	Glycine	0.92%
NDF	31.05%	Starch	3.03%	Threonine	0.63%
Lysine	0.98%	Methionine	0.39%	Manganese	≥30 mg/kg (ppm)
Histidine	0.42%	Arginine	0.91%	Phosphorus	0.69%
Crude fat (min)	1.0%	Cysteine	0.31%	Potassium	3.68%
Crude fiber (max)	18.5%	Leucine	1.05%	Calcium	≥0.4%
Ash (max)	12.0%	Phenylalanine	0.65%	Iron	≥250 mg/kg (ppm)

Note: DVAQUA^®^ postbiotic is available from Diamond V Mills Inc., Cedar Rapids, IA, USA, on www.diamondv.com (accessed on 9 April 2025). Abbreviations: ADF, acid detergent fiber; NDF, neutral detergent fiber.

**Table 3:** Primers used in the current study for qPCR.

Primer	Sequence (5′-3′)	Amplicon Size	Accession Number	Efficiency	References
*EF1-α*-F *EF1-α*-R	CTACGTGACCATCATTGATGCC AACACCAGCAGCAACGATCA	106	AB075952.1	1.917	[53]
*β-actin* F *β -actin* R	CAGCAAGCAGGAGTACGATGAG TGTGTGGTGTGTGGTTGTTTT	62	MX003455949	1.931	[52]
*IGF-1*-F *IGF-1*-R	GTCTGTGGAGAGCGAGGCTTT CACGTGACCGCCTTGCA	70	NM_001279503	1.945	[47]
*SOD2*-F *SOD2*-R	CTCCAGCCTGCCCTCAA TCCAGAAGATGGTGTGGTTAATGTG	58	XM_003449940.3	1.929	[48]
*IL-1 β* F *IL-1 β* R	TGCACTGTCACTGACAGCCAA ATGTTCAGGTGCACTATGCGG	113	DQ061114.1	1.913	[49]
*IL-10* F *IL-10* R	CTGCTAGATCAGTCCGTCGAA GCAGAACCGTGTCCAGGTAA	94	XM_003441366.2	1.9.37	[50]
*IgM F* *IgM R*	AGGAGACAGGACTGGAATGCACAA GGAGGCAGTATAGGTATCATCCTC	171	KJ676389.1	1.959	[51]

**Table 4:** Effects of *Saccharomyces cerevisiae* postbiotic on feed efficiency and growth performance of Nile tilapia at 30 and 60 days after the commencement of the feeding experiment.

Parameter	30 d				60 d			
	C	T1	T2	T3	C	T1	T2	T3
IBW (g)	35.1 ± 0.9	35.1 ± 0.9	35.1 ± 0.9	35.1 ± 0.9	35.1 ± 0.9	35.1 ± 0.9	35.1 ± 0.9	35.1 ± 0.9
FBW (g)	41.9 ± 0.4 ^d^	49.1 ± 1.2 ^cd^	55.3 ± 0.8 ^c^	64.8 ± 1.8 ^bc^	61.6 ± 0.3 ^bc^	71.1 ± 1.4 ^b^	84.1 ± 1.9 ^a^	94.5 ± 1.7 ^a^
WG (g/fish)	6.7 ± 0.6 ^d^	14.0 ± 0.5 ^cd^	20.2 ± 0.4 ^c^	29.7 ± 1.0 ^bc^	26.5 ± 0.6 ^bc^	36.0 ± 0.5 ^b^	49.0 ± 0.1 ^a^	59.4 ± 0.9 ^a^
%WG	24.4 ± 1.7 ^f^	41.6 ± 1.1 ^f^	62.5 ± 1.8 ^e^	85.2 ± 1.4 ^d^	84.6 ± 3.1 ^d^	106.1 ± 0.1 ^c^	141.8 ± 1.1 ^b^	175.6 ± 2.5 ^a^
FI (g/fish)	31.9 ± 0.2 ^f^	32.7 ± 0.3 ^ef^	35.1 ± 0.8 ^def^	37.7 ± 1.2 ^de^	42.6 ± 0.4 ^cd^	47.8 ± 1.3 ^bc^	53.2 ± 1.4 ^b^	61.3 ± 1.2 ^a^
FCR	4.3 ± 0.2 ^a^	2.5 ± 0.1 ^ab^	1.9 ± 0.1 ^bc^	1.3 ± 0.1 ^bc^	2.1 ± 0.1 ^bc^	1.4 ± 0.0 ^bc^	1.0 ± 0.0 ^bc^	0.8 ± 0.0 ^c^
GR (g/d)	0.2 ± 0.0 ^e^	0.5 ± 0.0 ^de^	0.7 ± 0.0 ^cd^	1.0 ± 0.0 ^bcd^	0.9 ± 0.0 ^bc^	1.2 ± 0.0 ^b^	1.6 ± 0.0 ^a^	2.0 ± 0.0 ^a^
SGR (%/d)	0.3 ± 0.0 ^e^	0.5 ± 0.0 ^de^	0.7 ± 0.0 ^cd^	0.9 ± 0.0 ^bcd^	0.9 ± 0.0 ^bc^	1.0 ± 0.0 ^b^	1.3 ± 0.0 ^a^	1.5 ± 0.0 ^a^
Survival (%)	100.0 ± 0.0	100.0 ± 0.0	100.0 ± 0.0	100.0 ± 0.0	100.0 ± 0.0	100.0 ± 0.0	100.0 ± 0.0	100.0 ± 0.0

Data are represented as means ± SEM. Means with different superscripts indicate that the corresponding means differ significantly at *p* ≤ 0.05 according to two-way ANOVA. Abbreviations: **C**, control: fish fed on a basal diet that contains no postbiotics; **T1**, treatment one: fish fed on a diet containing 1.25 g of *S. cerevisiae* postbiotic per kg fish feed; **T2**, treatment two: fish fed on a diet containing 2.5 g of *S. cerevisiae* postbiotic per kg fish feed; **T3**, treatment three: fish fed on a diet containing 5 g of *S. cerevisiae* postbiotic per kg fish feed; IBW, initial body weight; FBW, final body weight; WG, weight gain; %WG, weight gain percent; FI, feed intake; FCR, feed conversion ratio; GR, growth rate; SGR, specific growth rate.

**Table 5:** Impact of *Saccharomyces cerevisiae* postbiotic on the serum biochemical parameters of Nile tilapia 30 and 60 days after the start of the feeding experiment.

Parameter	30 d				60 d			
	C	T1	T2	T3	C	T1	T2	T3
TP (g/dL)	2.2 ± 0.1 ^f^	2.7 ± 0.6 ^def^	3.4 ± 0.2 ^bc^	3.9 ± 0.2 ^b^	2.7 ± 0.1 ^e^	3.1 ± 0.2 ^cd^	4.3 ± 0.7 ^abc^	4.6 ± 0.3 ^a^
ALB (g/dL)	1.1 ± 0.1 ^c^	1.1 ± 0.5 ^c^	1.3 ± 0.4 ^bc^	1.5 ± 0.1 ^ab^	1.2 ± 0.1 ^c^	1.2 ± 0.0 ^c^	1.3 ± 0.2 ^ac^	1.5 ± 0.0 ^a^
GLOB (g/dL)	1.1 ± 0.1 ^f^	1.6 ± 0.6 ^de^	2.1 ± 0.2 ^bc^	2.4 ± 0.2 ^bc^	1.5 ± 0.1 ^ef^	1.9 ± 0.2 ^cd^	3.1 ± 0.6 ^ab^	3.1 ± 0.3 ^a^
A/G ratio	1.2 ± 0.2 ^a^	0.7 ± 0.2 ^ab^	0.6 ± 0.0 ^b^	0.7 ± 0.1 ^bcd^	1.0 ± 0.1 ^ac^	0.7 ± 0.1 ^b^	0.5 ± 0.1 ^e^	0.5 ± 0.0 ^de^
AST (U/L)	155.6 ± 4.9	151.1 ± 5.1	147.9 ± 5.6	140.2 ± 4.1	153.8 ± 2.5	149.1 ± 3.9	144.7 ± 6.3	140.6 ± 5.8
Creat (mg/dL)	0.5 ± 0.1 ^a^	0.3 ± 0.1 ^b^	0.2 ± 0.1 ^b^	0.2 ± 0.0 ^b^	0.4 ± 0.0 ^a^	0.2 ± 0.0 ^b^	0.2 ± 0.0 ^b^	0.2 ± 0.0 ^b^
GLU (mg/dL)	116.2 ± 5.0 ^a^	106.2 ± 1.6 ^b^	94.2 ± 6.0 ^c^	76.1 ± 2.5 ^d^	119.3 ± 3.8 ^a^	98.3 ± 5.6 ^bc^	91.8 ± 1.9 ^c^	72.9 ± 3.9 ^d^

Abbreviations: TP: total protein, ALB: albumin, GLOB: globulin, A/G: albumin/globulin ratio, AST: aspartate aminotransferase, Creat: creatinine, GLU: glucose. Data are represented as means ± SEM. Within rows, values with different superscripts indicate that their corresponding means differ significantly at *p* ≤ 0.05.

## Data Availability

The original contributions presented in this study are included in the article. The strain used in the current study is available in GenBank under accession number PZ346033 (*Fusarium oxysporum* strain TB-ESHA-3 small subunit ribosomal RNA gene, -Nucleotide-NCBI). Further inquiries for raw data can be directed to the corresponding authors.

## Supplementary Materials

The following supporting information can be downloaded at https://www.mdpi.com/article/10.3390/biology15171445/s1, Figure S1. Heatmap illustration of the expression of *IL-1β*, *IL-10*, *IGF-1*, and *SOD2* genes in the livers of Nile tilapia fed varying concentrations of *Saccharomyces cerevisiae* postbiotic at 30 and 60 days. Different letters indicate significant differences at *p* < 0.05. Figure S2. Heatmap displaying hierarchical clustering of the four genes, *IL-1β*, *IL-10*, *IGF-1*, and *SOD2*, in the liver of Nile tilapia after the administration of different concentrations of *Saccharomyces cerevisiae* postbiotic at 30 and 60 days after the start of the feeding experiment. Figure S3. The heatmap illustrates results from quantitative real-time PCR of mRNAs for immune-related genes (*IL-1β*, *IL-10*, and *IgM*) in the spleen of Nile tilapia (*Oreochromis niloticus*) at two distinct experimental intervals (30 and 60 days) following the start of the feeding experiment, following the administration of varying concentrations of *Saccharomyces cerevisiae* postbiotic. Different letters indicate significant differences at *p* < 0.05. Figure S4. Heatmap displaying column hierarchical clustering of the three genes (*IL-1β*, *IL-10*, and *IgM*) in the spleen of Nile tilapia (*Oreochromis niloticus*) at two distinct experimental intervals (30 and 60 days) after the start of the feeding experiment, following the administration of varying concentrations of *Saccharomyces cerevisiae* postbiotic. The values of row expressions are shown using a colour scale ranging from blue for lower expression to yellow for higher expression. Figure S5. The heatmap illustrates results from quantitative real-time PCR of mRNAs for immune-related genes (*IL-1β, IL-10*, and *IgM*) in the anterior intestine of Nile tilapia (*Oreochromis niloticus*) after feeding on different concentrations of *Saccharomyces cerevisiae* postbiotic over two different checkpoints (30 and 60 d) post-feeding commencement. Different letters indicate a significant difference at *p* < 0.05. Figure S6. Heatmap displaying column hierarchical clustering of the three genes (*IL-1β, IL-10*, and *IgM)* in the anterior intestine of Nile tilapia after receiving different concentrations of *Saccharomyces cerevisiae* postbiotic over two different experimental times (30 and 60 days) post-feeding experiment commencement. Figure S7. The heatmap illustrates results of maximum fold change of the *IL-1β* gene in all examined organs of Nile tilapia (*Oreochromis niloticus*) following two distinct experimental intervals (30 and 60 days) after the start of the feeding experiment, following the administration of varying dosages of *Saccharomyces cerevisiae* postbiotic. Different letters indicate significant differences at *p* < 0.05. Figure S8. The heatmap illustrates results of maximum fold change of the *IL-10* gene in all examined organs of Nile tilapia (*Oreochromis niloticus*) following two distinct experimental intervals (30 and 60 days) after the start of the feeding experiment, following the administration of varying dosages of *Saccharomyces cerevisiae* postbiotic. Different letters indicate significant differences at *p* < 0.05. Figure S9. The heatmap illustrates results of maximum fold change of the *IgM* gene in all examined organs of Nile tilapia (*Oreochromis niloticus*) following two distinct experimental intervals (30 and 60 days) after the start of the feeding experiment, following the administration of varying dosages of *Saccharomyces cerevisiae* postbiotic. Data are presented as the means (±SEM). Different lowercase letters indicate significant differences (*p* < 0.05).

## Abbreviations

The following abbreviations are used in this manuscript:

Ash	Ash content
C	Control group
DE	Digestible energy
FCR	Feed conversion ratio
FI	Feed intake
WG	Weight gain
SGR	Specific growth rate
Ln	Natural logarithm
GR	Growth rate
CLRs	C-type lectin receptors
CR3	Complement Receptor Type 3
PRRs	Pattern recognition receptors
NK cells	Natural killer cells
IBW	Initial body weight
FBW	Final body weight
TP	Total protein
ALB	Albumin
GLOB	Globulin
A/G	Albumin/globulin ratio
AST	Aspartate aminotransferase
Creat	Creatinine
GLU	Glucose
SEM	Standard error of the mean
ANOVA	Analysis of variance
RT-PCR	Reverse transcription polymerase chain reaction
RT-qPCR	Quantitative real-time PCR
cDNA	Complementary DNA
*IGF-1*	Insulin-like Growth Factor-1
*SOD2*	Superoxide Dismutase 2
*IL-1β*	Interleukin-1 beta
*IL-10*	Interleukin-10
*IgM*	Immunoglobulin M
EF1α	Elongation factor 1 alpha
β-actin	Beta- actin
*IL-8*	Interleukin 8
*TNF-α*	Tumer necrosis factor -alpha
*IFN-γ*	Interferone-gamma
Ct	Cycle threshold
2^−(ΔΔCt)^	Delta-delta Ct fold-change
BG	Beta-glucan
MOS	Mannan-oligosaccharides
SGLT-1	Sodium-glucose linked transporter 1
TLRs	Toll-like receptors
TNF	Tumor necrosis factor
DM	Dry matter
CP	Crude protein
CF	Crude fiber
EE	Ether extract
NFE	Nitrogen-free extract
ADF	Acid detergent fiber
NDF	Neutral detergent fiber
mg/kg (ppm)	Milligrams per kilogram (parts per million)
g/dL	Grams per deciliter
mg/dL	Milligrams per deciliter
U/L	Units per liter
°C	Degrees Celsius
Mm	Millimeter
Mm	Micrometer
Mg	Microgram
Ml	Microliter
L	Liter
ISAPP	International Scientific Association for Probiotics and Prebiotics
T1	Treatment 1 (1.25 g/kg DVAQUA^®^)
T2	Treatment 2 (2.5 g/kg DVAQUA^®^)
T3	Treatment 3 (5 g/kg DVAQUA^®^)
