# Supplementary material for: Effects of Saccharomyces cerevisiae Postbiotic on Growth Performance, Selected Immune and Antioxidant Gene Expression, and Resistance to Fusarium oxysporum Infection in Nile Tilapia (Oreochromis niloticus)
Source: Biology (Basel). 2026 Aug 22;15(17):1445. doi: 10.3390/biology15171445 (PMC13565351; doi:10.3390/biology15171445)
Supplement: Supplementary file 1 [file biology-15-01445-s001.zip › biology-4483403-supplementary.pdf]

Effects of *Saccharomyces cerevisiae* postbiotic on growth performance, selected immune and antioxidant gene expression, and resistance to *Fusarium oxysporum* infection in Nile tilapia (*Oreochromis niloticus*)

Nagwa Mohamed Abdou Romeih [1], Mahmoud Mostafa Mahmoud [1,2,*], Yosra M. I. El Sherry [3], Amna Mohamed Eltayeb [4], Amr Abdallah [5], Abeer M. Mahmoud [6,7,*], Marco Albano [8], Alaa G. M. Osman [9], Ebtsam Sayed Hassan Abdallah [1]

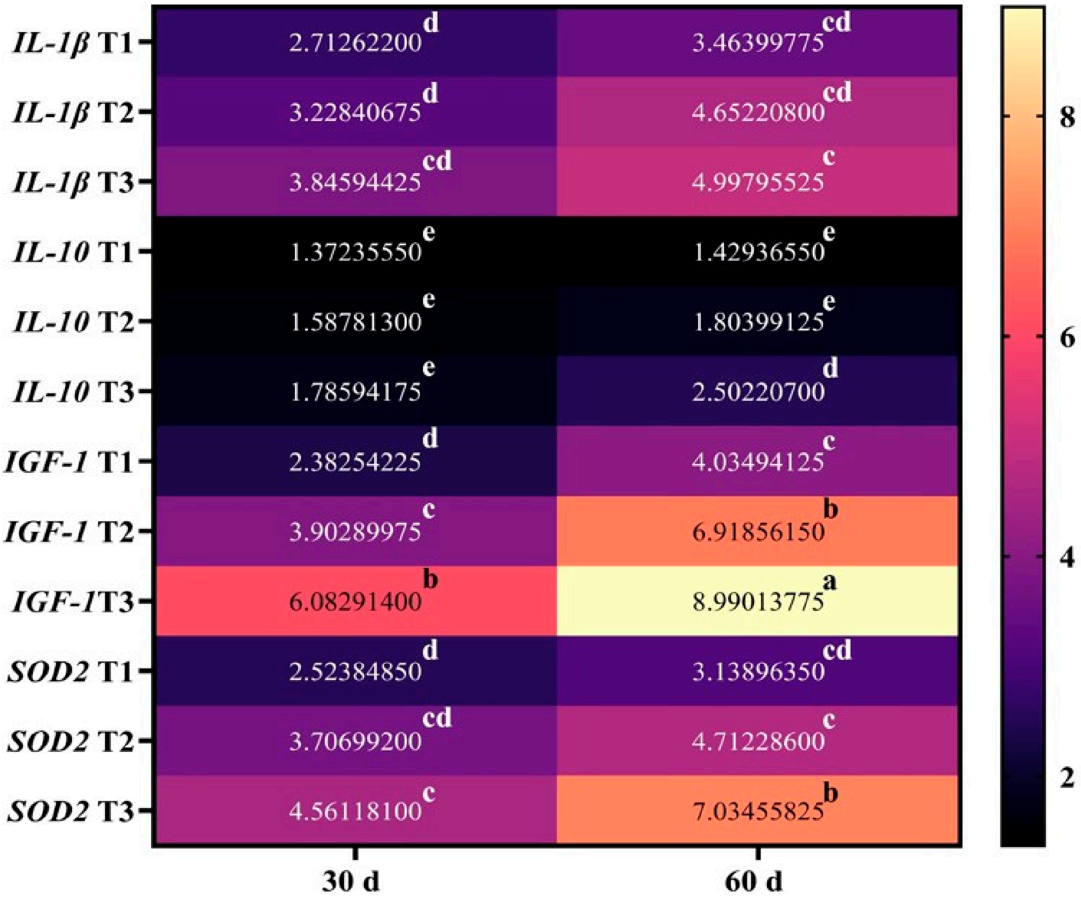

Figure S1. Heatmap illustration of the expression of *IL-1β*, *IL-10*, *IGF-1*, and *SOD2* genes in the livers of Nile tilapia fed varying concentrations of *Saccharomyces cerevisiae* postbiotic at 30 and 60 days. Different letters indicate significant differences at $p < 0.05$.

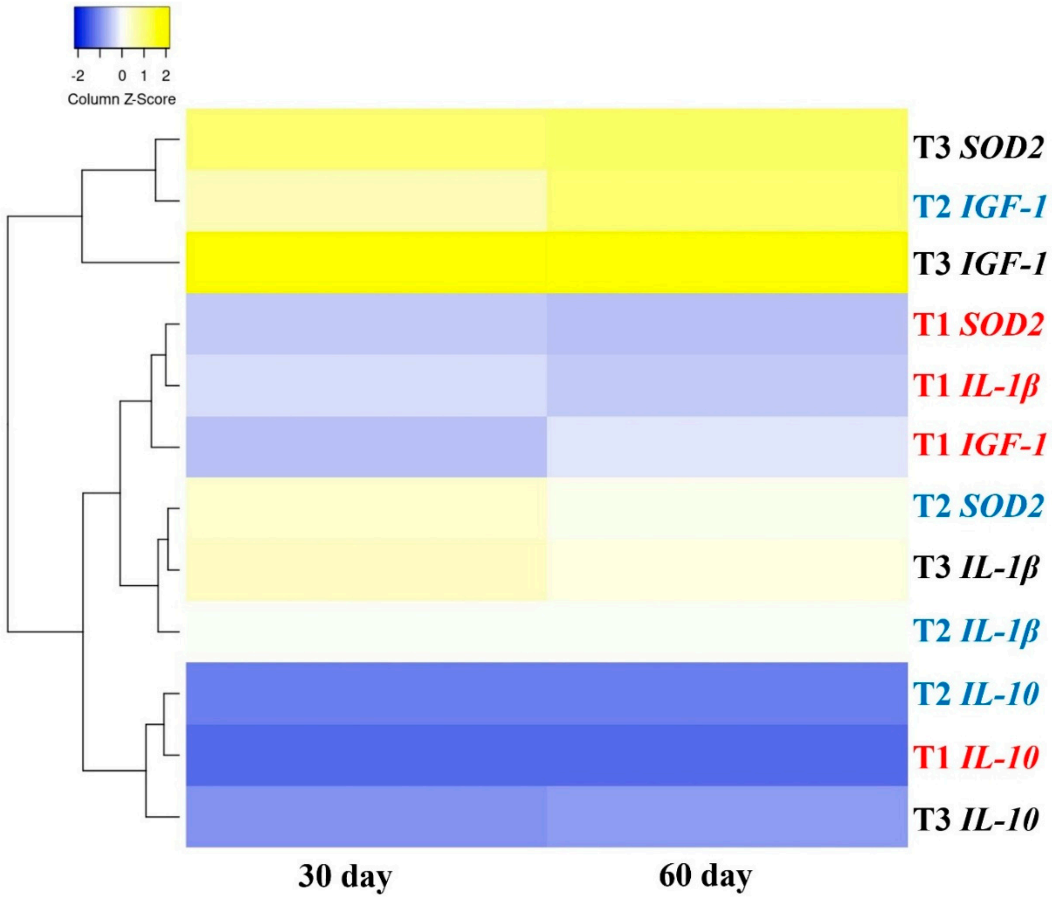

Figure S2. Heatmap displaying hierarchical clustering of the four genes, *IL-1β*, *IL-10*, *IGF-1*, and *SOD2*, in the liver of Nile tilapia after the administration of different concentrations of *Saccharomyces cerevisiae* postbiotic at 30 and 60 days after the start of the feeding experiment.

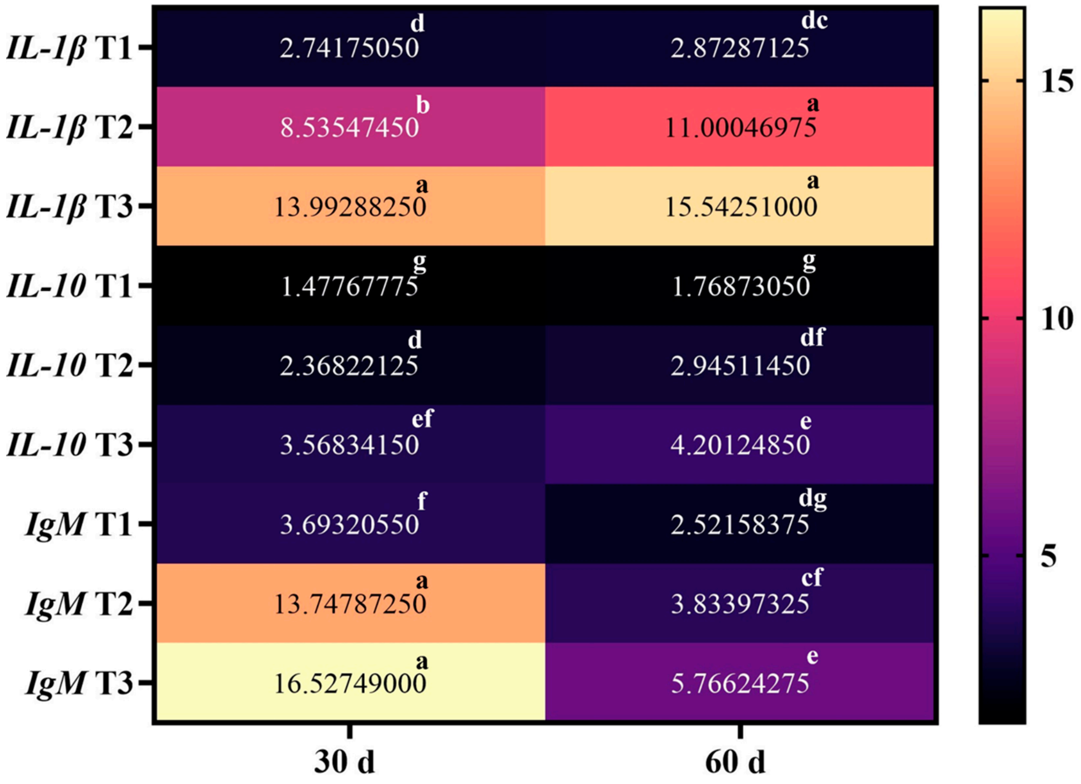

Figure S3. The heatmap illustrates results from quantitative real-time PCR of mRNAs for immune-related genes (*IL-1β*, *IL-10*, and *IgM*) in the spleen of Nile tilapia (*Oreochromis niloticus*) at two distinct experimental intervals (30 and 60 days) following the start of the feeding experiment, following the administration of varying concentrations of *Saccharomyces cerevisiae* postbiotic. Different letters indicate significant differences at $p < 0.05$.

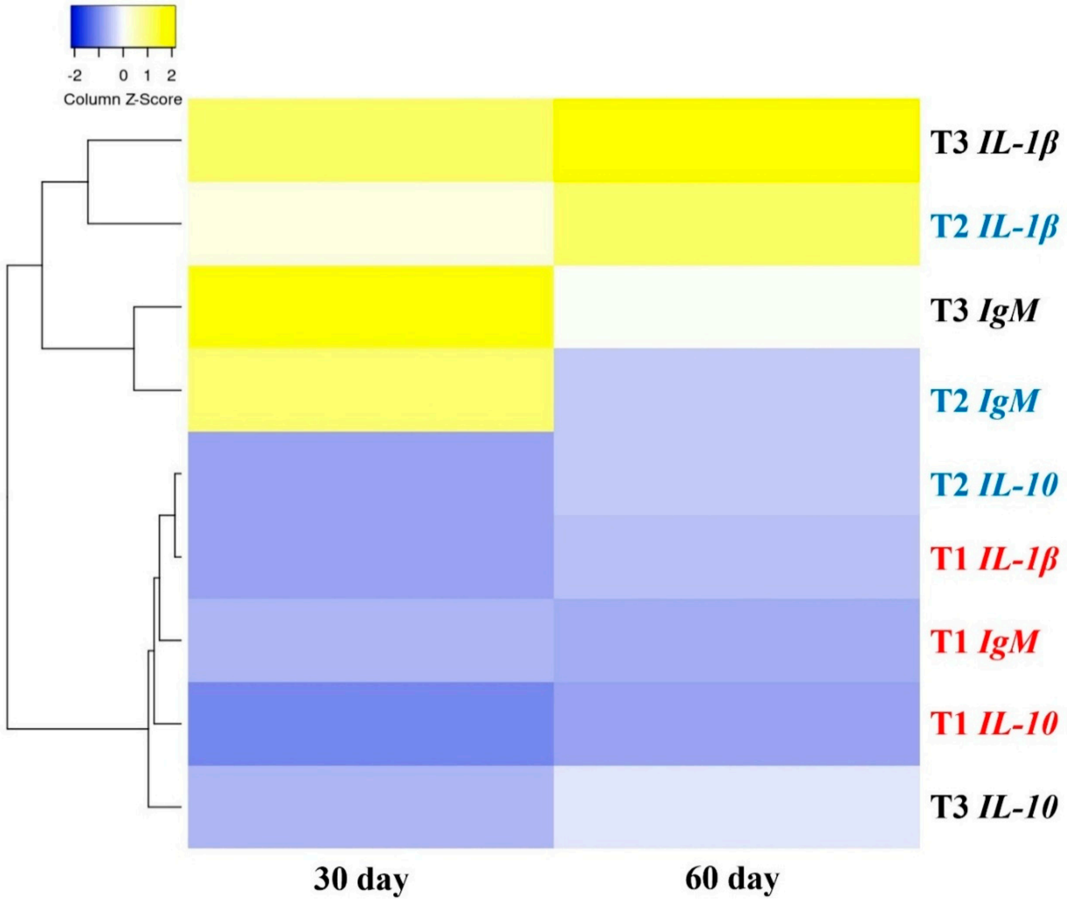

Figure S4. Heatmap displaying column hierarchical clustering of the three genes (*IL-1β*, *IL-10*, and *IgM*) in the spleen of Nile tilapia (*Oreochromis niloticus*) at two distinct experimental intervals (30 and 60 days) after the start of the feeding experiment, following the administration of varying concentrations of *Saccharomyces cerevisiae* postbiotic. The values of row expressions are shown using a colour scale ranging from blue for lower expression to yellow for higher expression.

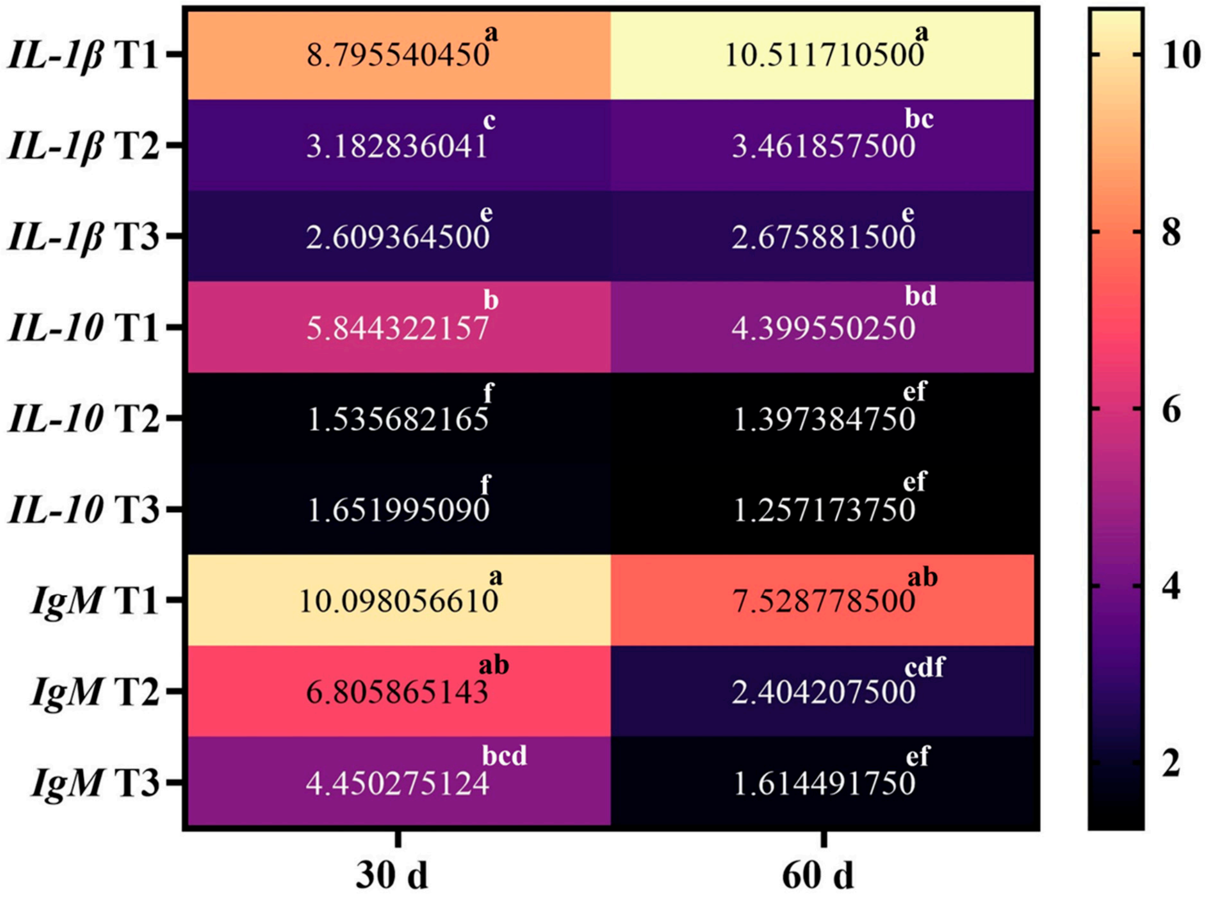

Figure S5. The heatmap illustrates results from quantitative real-time PCR of mRNAs for immune-related genes (*IL-1β*, *IL-10*, and *IgM*) in the anterior intestine of Nile tilapia (*Oreochromis niloticus*) after feeding on different concentrations of *Saccharomyces cerevisiae* postbiotic over two different checkpoints (30 and 60 d) post-feeding commencement. Different letters indicate a significant difference at $p < 0.05$.

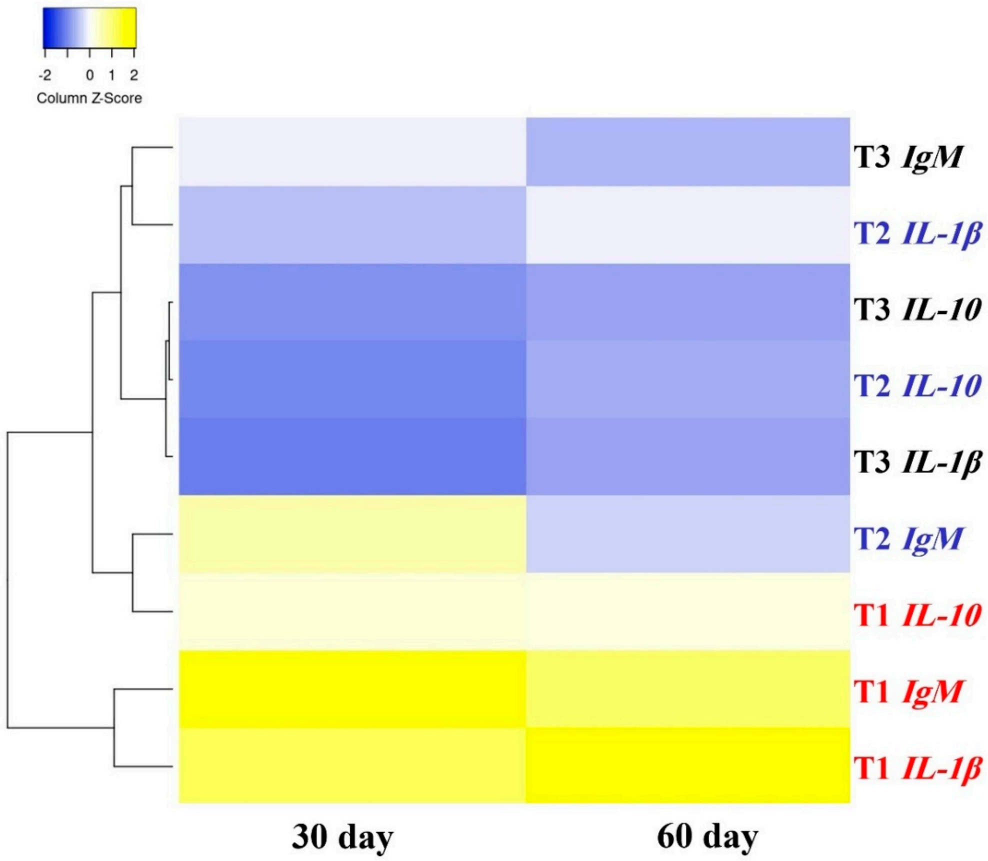

Figure S6. Heatmap displaying column hierarchical cluster of the three genes (*IL-1β, IL-10*, and *IgM)* in the anterior intestine of Nile tilapia after receiving different concentrations of *Saccharomyces cerevisiae* postbiotic over two different experimental times (30 and 60 days) post-feeding experiment commencement.

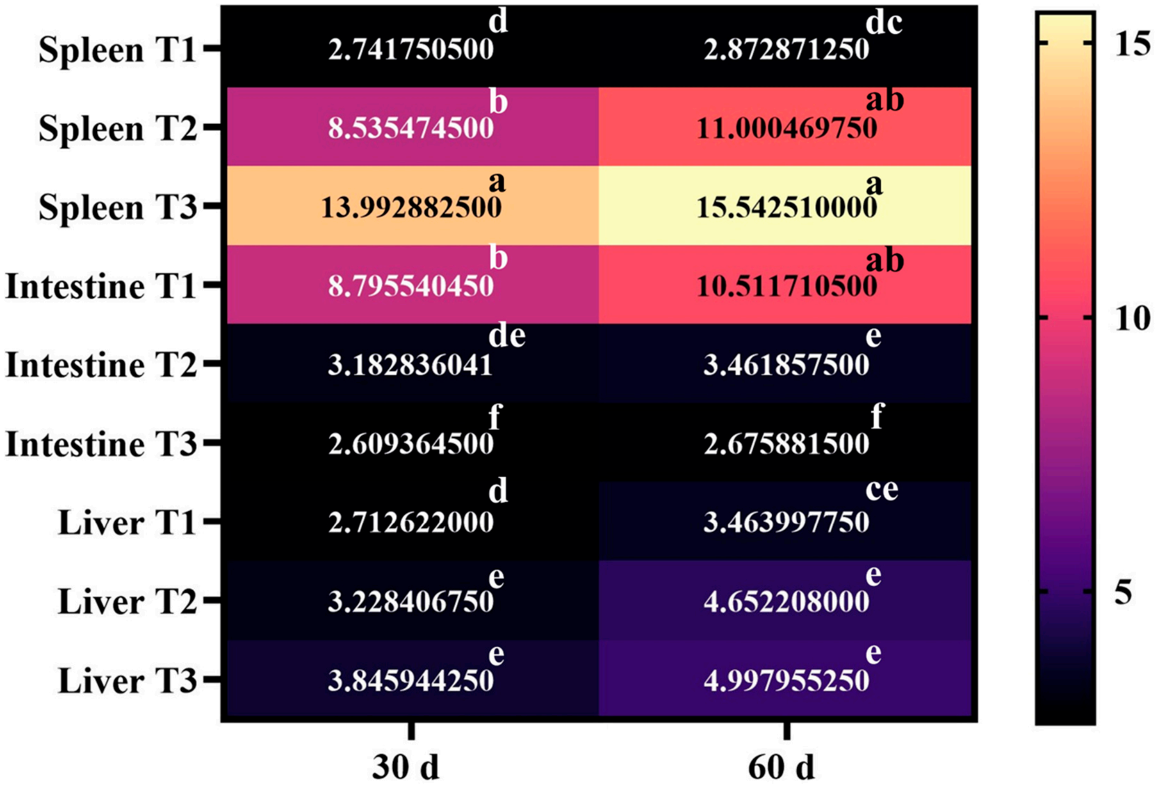

Figure S7. Heatmap illustrates results of maximum fold change of the *IL-1β* gene in all examined organs of Nile tilapia (*Oreochromis niloticus*) following two distinct experimental intervals (30 and 60 days) following the start of the feeding experiment, following the administration of varying dosages of *Saccharomyces cerevisiae* postbiotic. Different letters indicate significant differences at $p < 0.05$.

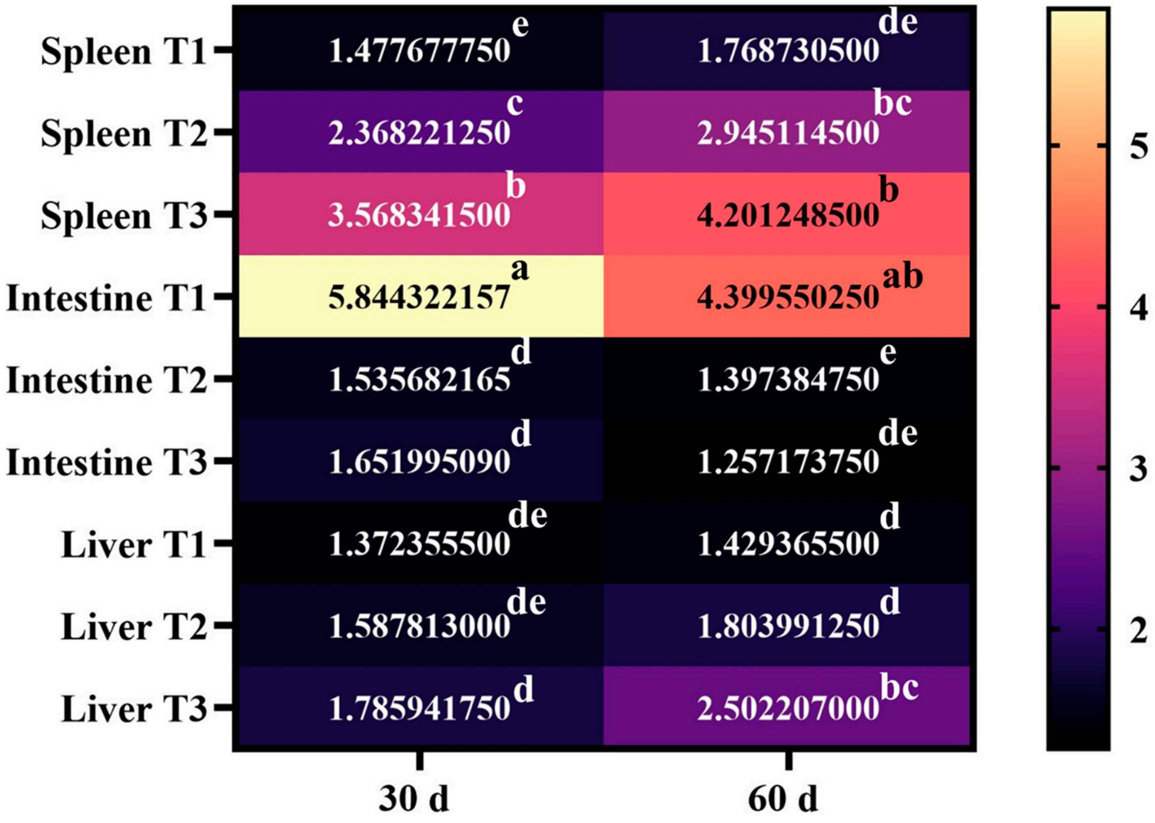

Figure S8. Heatmap illustrates results of maximum fold change of the *IL-10* gene in all examined organs of Nile tilapia (*Oreochromis niloticus*) following two distinct experimental intervals (30 and 60 days) following the start of the feeding experiment, following the administration of varying dosages of *Saccharomyces cerevisiae* postbiotic. Different letters indicate significant differences at $p < 0.05$.

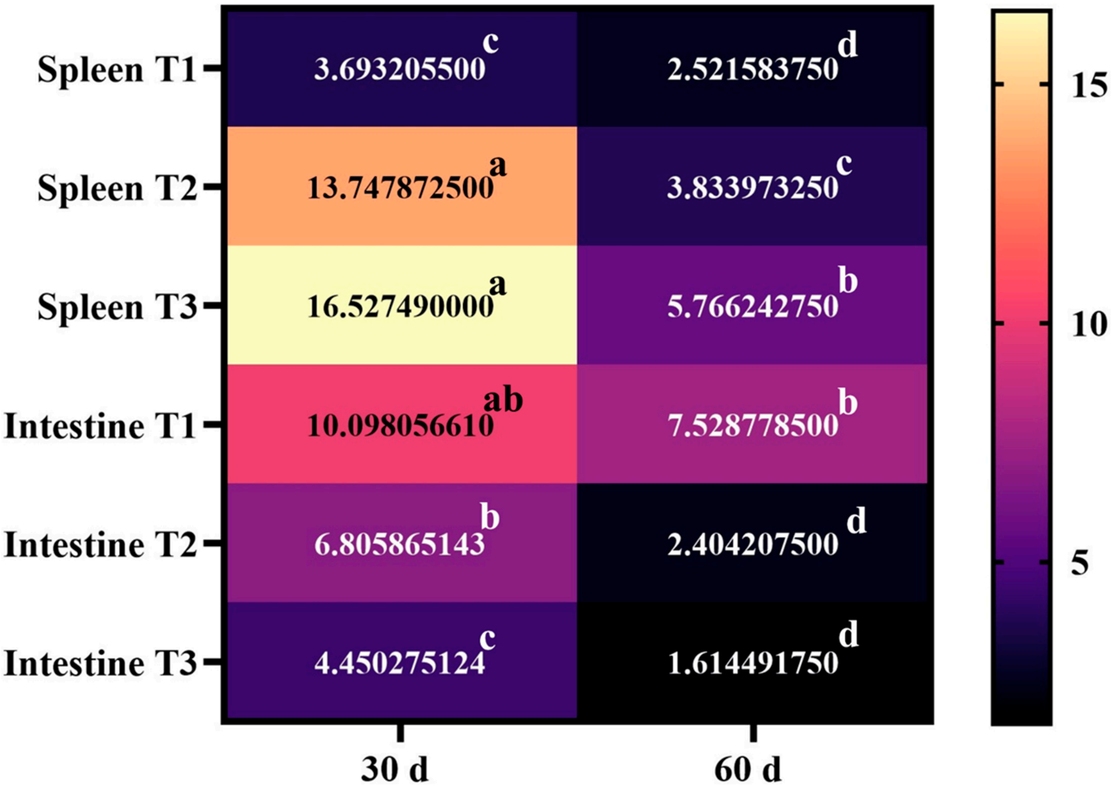

Figure S9. Heatmap illustrates results of maximum fold change of the *IgM* gene in all examined organs of Nile tilapia (*Oreochromis niloticus*) following two distinct experimental intervals (30 and 60 days) after the start of the feeding experiment, following the administration of varying dosages of *Saccharomyces cerevisiae* postbiotic. Data are presented as the means (± SEM). Different lowercase letters indicate significant differences ($p < 0.05$)
